# Physiological Responses and Metabolic Characteristics of Proso Millet Under Drought Stress During Germination Period

**DOI:** 10.1002/fsn3.70001

**Published:** 2025-01-31

**Authors:** Mengyao Wang, Yulu Hu, Jiao Mao, Yuanmeng Xu, Shu Wang, Lun Wang, Zhijun Qiao, Sichen Liu, Xiaoning Cao

**Affiliations:** ^1^ Center for Agricultural Genetic Resources Research Shanxi Agricultural University Taiyuan China; ^2^ College of Agriculture Shanxi Agricultural University Jinzhong China

**Keywords:** amino acid, budding, flavonoids, phenolic acids, proso millet

## Abstract

To clarify the impact of drought stress during germination on proso millet's physiological responses and metabolic features, this study used physiological and targeted‐like metabolomics methods. With Longmi No. 7 (drought‐tolerant, L1) and Longmi No. 15 (drought‐sensitive, L2) as materials, we studied the enzyme activities, osmotic adjustment substances, and differential metabolites of proso millet. Results showed that under drought stress, L1's enzyme activities and osmotic adjustment substance contents were significantly higher than L2's, especially at 48‐h treatment. 1085 known metabolites were identified from 24 samples, under normal germination, L1's main differential metabolites (amino acids, flavonoids, phytohormone, lipids, sugars, etc.) were enriched in amino acid, lipid, sugar, and energy metabolism pathways. L2's (amino acids, sugars, flavonoids, etc.) were in sugar, lipid metabolism, secondary metabolite biosynthesis, and amino acid metabolism pathways. At 24‐h treatment, the metabolic pathways of L1 were mainly concentrated in carbohydrate and nucleotide metabolism, while those of L2 were mainly in carbohydrate and lipid metabolism. At 48 h, the metabolic pathways of L1 were mainly in carbohydrate, energy and lipid metabolism, and those of L2 were mainly in carbohydrate, lipid metabolism, biosynthesis of other secondary metabolites and amino acid metabolism. Under stress, L1's main differential metabolites were organic acids, sugars, flavonoids, amino acids, etc.; L2's were phytohormones, organic acids, sugars, flavonoids, amino acids. This study provides a new direction for the development of proso millet sprouts. Meanwhile, it offers new ideas and theoretical bases for the development of functional foods and the regulation of nutritional components of proso millet.

## Graphical abstract

During the germination process of proso millet, the differential metabolites are mainly amino acids, sugars, and flavonoids, etc., and they are mainly enriched in amino acid, lipid, sugar, and energy metabolism pathways. Under drought‐stress treatment, the differential metabolites of proso millet are mainly organic acids, flavonoids, amino acids, and sugars. They are mainly enriched in the biosynthesis of secondary metabolites, tyrosine metabolism, and ABC transporter pathways. The physiological and metabolic characteristics of proso millet sprouts under drought stress have been revealed, thereby clarifying their nutritional and functional characteristics.

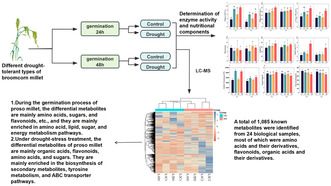

## 1 Introduction

Proso millet ( *Panicum miliaceum* L.) is a short‐day C_4_ crop (Nielsen and Vigil 2017), characterized by a short growth period, drought tolerance, and tolerance to barrenness (Diao 2017). It is widely cultivated in the arid and semi‐arid regions of northern China (Wang, Mao et al. 2024), and is rich in various nutrients such as dietary fiber, antioxidants, phytochemicals, and polyphenols (Muthamilarasan et al. 2016). It is gluten‐free and has a low glycemic index (Marti and Tyl 2021), providing an ideal food choice for people with special dietary needs (such as those with gluten intolerance and diabetes patients), and has significant importance and wide application in food safety and health promotion (Vidhya et al. 2023; Singh et al. 2023).

The germination period is a crucial stage in the plant life cycle. During the germination process, seed metabolism is reactivated, producing amino acids, reducing sugars, small dextrins, different organic acids, and other compounds (Jiménez et al. 2024). This process promotes the expression of genes related to hydrolytic enzymes (Ren, Hong et al. 2024), and then leads to an increase in bioactive substances such as soluble dietary fiber and phenolic compounds in the cereal cortex, improving the nutritional and processing properties of the grain (Gao et al. 2024). During the germination process, minerals are released and become more easily absorbed by the intestine, and at the same time, vitamins and γ‐aminobutyric acid (GABA) are synthesized and accumulated (Lemmens et al. 2019). The intake of germinated millet has outstanding effects on controlling blood pressure, regulating blood sugar concentration, and preventing chronic diseases such as colorectal cancer (Feng et al. 2020). Yang et al. (2021) found that proso millet sprout powder increased the activity of α‐amylase and the contents of crude fiber, soluble sugar, free amino acids, and bioactive components, and increased the contents of flavonoids, phenols, and antioxidants (Chen et al. 2017), optimized the nutritional structure of proso millet, and significantly improved its sensory quality and flavor characteristics (Ding et al. 2011), making sprouts gradually favored in the market and becoming a healthy food choice that combines high nutritional value, economy, and environmental protection (Wang et al. 2022).

Adequate water supply is an important factor affecting the normal germination, yield, and quality formation of sprouts. Compared with sensitive varieties, drought‐tolerant varieties show enhanced water absorption capacity, rapid germination, and accelerated radicle growth rate, promoting the rapid establishment of the seedling root system (Wang et al. 2021), thereby increasing the yield of sprouts and laying a good foundation for subsequent growth and development. Water deficiency will cause a series of complex changes in crop morphological structure, physiological and biochemical aspects of cells and molecules (Oguz et al. 2022), including a reduction in the root‐shoot ratio, the accumulation of proline, and the production of reactive oxygen species (Lal et al. 2022), and reactive oxygen species can cause damage to cell structure, proteins, lipids, carbohydrates, and nucleic acids, leading to cell death (Uzilday et al. 2012), thereby reducing the quality of sprouts. To cope with drought stress, plants have evolved a series of adaptive mechanisms, such as changing the morphological structure of crops (Fang and Xiong 2015), the synthesis of ABA in plant organs and the regulation of stomatal conductance and related gene expression (Lim et al. 2015), and the protection of cell functions by proline through scavenging reactive oxygen species (Kaur and Asthir 2015). Water changes also affect the metabolic pathways of crops. When water is deficient, proso millet seedlings may maintain the stability of cell osmosis and reduce water loss by promoting lignin metabolism and fatty acid biosynthesis (Cui et al. 2023); enhance the active synthesis metabolism of nutrients, improve ROS detoxification ability, osmotic adjustment ability, and membrane stability (Guo et al. 2018); promote the galactose metabolism pathway and the TCA cycle to provide material and energy support for the biosynthesis of aminoacyl‐tRNA and the metabolism of nicotinate and nicotinamide (Zhi et al. 2024).

At present, the research on proso millet germination mainly focuses on aspects such as enzyme activity and antioxidant activity (Yuan et al. 2024), as well as nutritional value and in vitro digestion characteristics (Yang et al. 2021), while the physiological responses and metabolic characteristics of different drought‐tolerant proso millet during the germination process are still unclear. In this study, using physiological and targeted‐like metabolomics techniques, the physiological characteristics and metabolic differences of different drought‐tolerant proso millet during the germination period were analyzed to clarify the germination metabolism mechanism of proso millet, providing a scientific basis for precisely regulating the proso millet germination process and improving the taste quality of sprouts, and being of great significance for the cultivation of special‐purpose proso millet varieties and promoting their standardized production.

## 2 Materials and Methods

### 2.1 Materials

In this study, proso millet varieties Longmi No. 7 (drought‐resistant variety, L1) and Longmi No. 15 (non‐drought‐resistant variety, L2) with different drought tolerance were used as experimental materials (Hu et al. 2023), which were provided by the Agricultural Gene Resources Research Center of Shanxi Agricultural University.

### 2.2 Experimental Design

Two treatment concentration control groups (deionized water) and treatment groups (20% PEG‐6000 (W/V) solution) were set up (Zhang et al. 2013), and two germination times were 24 h (C1, D1) and 48 h (C2, D2), as detailed in Table 1. The proso millet seeds (50 grains) were used for the germination experiment, with three replicates for each treatment. Samples were collected at 24 h and 48 h respectively, labeled, treated with liquid nitrogen, and stored in a refrigerator at −80°C.

**TABLE 1:** Experimental design.

Time	Longmei No. 7 (L1)		Longmei No. 15 (L2)	
	Control(C)	Drought(D)	Control(C)	Drought(D)
24 h	L1C1	L1D1	L2C1	L2D1
48 h	L1C2	L1D2	L2C2	L2D2

### 2.3 Test Indicators

#### 2.3.1 Determination of the Activities of SOD, POD and CAT Enzymes, and the Contents of Soluble Sugar, Proline, H₂O₂ and MDA

Weigh 0.1 g of sample, add 2 mL of phosphate buffer (50 mM, pH 7.8) and an appropriate amount of liquid nitrogen, grind evenly, centrifuge at 12000 × g and 4°C for 15 min, and take the supernatant to prepare enzyme solution for standby.

SOD was determined by the NBT photoreduction inhibition method. POD was determined by the guaiacol colorimetric method. CAT was determined by the ultraviolet absorption method. The content of soluble sugar was determined by the anthrone colorimetric method according to the method of Wang et al. (Wang et al. 2019). The content of soluble sugar was calculated according to the soluble sugar standard curve *y* = 0.0057*x* + 0.1785, *R* ^2^ = 0.9973. The content of proline was determined by the ninhydrin colorimetric method according to the method of Bates, Waldren, and Teare (1973). The mass fraction of proline in the test solution was obtained from the standard curve *y* = 0.0835*x* + 0.08, *R* ^2^ = 0.9973. Hydrogen peroxide (H_2_O_2_) was determined by a hydrogen peroxide kit (Beijing Solarbio). Malondialdehyde (MDA) was determined by a malondialdehyde kit (Beijing Solarbio), and the operation was strictly carried out in accordance with the kit instructions.

#### 2.3.2 Determination of Soluble Protein, Amylose and Total Starch Contents

The content of soluble protein was determined by the Coomassie Brilliant Blue G‐250 staining method according to the method of Bradford (1976). The concentration of soluble protein was obtained through the standard curve *y* = 0.0085*x* + 1.004, *R* ^2^ = 0.998. Amylose was determined by the colorimetric method according to the method of Chen et al. (2020). The amylose content in the sample was obtained from the standard curve *y* = 0.0845*x* + 0.004, *R* ^2^ = 0.9999. Total starch was determined by using a starch kit (Beijing Solarbio). The operations were strictly carried out in accordance with the kit instructions.

#### 2.3.3 Sample Preparation and Metabolome Data Acquisition

After grinding 100 mg of sample in liquid nitrogen, place it in an EP tube and add 500 μL of 80% methanol aqueous solution for extraction. After vortexing, standing on ice and two centrifugation treatments (15,000 × g, 4°C, 20 min), collect the supernatant and dilute it to 53% methanol content for subsequent analysis. At the same time, three quality control (QC) samples were prepared to monitor the analysis stability. The purified samples were loaded onto the LC–MS/MS system (equipped with ExionLC AD and QTRAP 6500+). Novogene Bioinformatics Technology Co. Ltd. (Beijing, China) extracted, detected and quantitatively analyzed the metabolites in the samples.

#### 2.3.4 Liquid chromatography, tandem mass spectrometry conditions and identification of metabolites

The chromatographic column is Xselect HSS T3 (2.5 μm, 2.1 × 150 mm), the column temperature is 50°C, the flow rate is 0.4 mL/min, and the mobile phases are A (0.1% formic acid‐water) and B (0.1% formic acid‐acetonitrile). Electrospray ionization detection is performed in positive and negative ion modes respectively. Finally, LC–MS/MS data is processed by SCIEX OS V1.4 software, metabolites are quantified by Q3, and metabolite identification is performed by combining information such as Q1, Q3, RT (retention time), DP (declustering potential) and CE (collision energy).

### 2.4 Statistical Analysis

One‐way analysis of variance (ANOVA) and Duncan's multiple range test (*p* < 0.05) were performed using SPSS 22.0 statistical software. Charts are drawn using OriginPro 2022 (OriginLab, Northampton, MA, USA). Principal component analysis (PCA) and partial least squares discriminant analysis (PLS‐DA) are performed on the Beijing Novogene cloud platform to obtain the VIP value of each metabolite. Based on the *t*‐test to calculate the statistical significance (P value) of each metabolite between the two groups, and calculate the fold change (FC value) of the metabolite between the two groups. Differential metabolites are screened according to *p* < 0.05 and |log_2_FC| > 1. The KEGG database (https://www.genome.jp/kegg/pathway.html) is used to study the function and metabolic pathways of metabolites. When *x*/*n* > *y*/*n*, it is considered that this metabolic pathway is enriched; when the P value of the metabolic pathway is < 0.05, it is considered that this metabolic pathway is significantly enriched. The rest of the charts are drawn using OriginPro 2022.

## 3 Results and Discussion

### 3.1 Changes in the Activities of SOD, POD, and CAT Enzymes and the Contents of MDA, Proline, and Soluble Sugar in Proso Millet Under Drought Stress During the Germination Period

**FIGURE 1:**
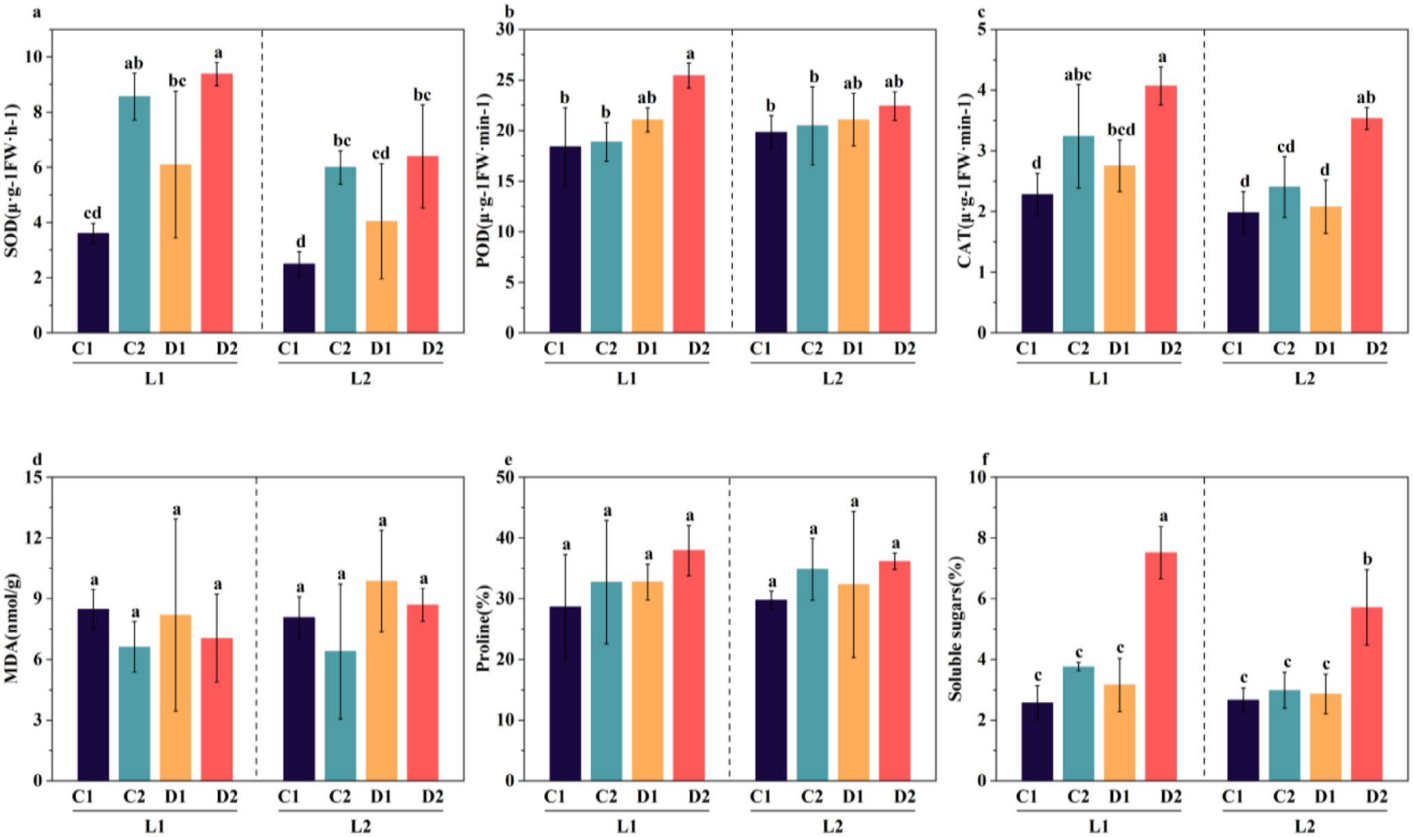
Changes in the activities of SOD, POD, and CAT enzymes and the contents of MDA, proline, and soluble sugar in different proso millet varieties at 24 h and 48 h under drought stress and normal germination. (a) SOD content; (b) POD content; (c) CAT content; (d) MDA content; (e) proline content; (f) soluble sugar content. These values are the averages of three replicates. Vertical bars indicate the mean standard deviation. Significance is indicated according to Duncan's multiple range test (*p* < 0.05).

Drought stress during the germination period has a significant impact on the activities of SOD, POD, and CAT and the contents of MDA, proline, and soluble sugar in proso millet. Compared with the control, the activities of SOD, POD, and CAT and the contents of MDA, proline, and soluble sugar in L1D1, L1D2, L2D1, and L2D2 all increased significantly (Figure 1). And except for MDA, the content of the other indicators in L1 is significantly higher than that in L2, and the content under 48‐h treatment is higher than that under 24‐h treatment. For MDA, the content of L2 under 48‐h treatment is significantly higher than that of L1. Consistent with the study by Awan et al. (2024), it may be because SOD, POD, and CAT can protect cells from the negative effects of ROS under oxidative stress Li, Li et al. (2020b). SOD can quickly disproportion O₂⁻ into peroxides such as H₂O₂ and O₂ (Ru et al. 2023), and CAT can catalyze the decomposition of H₂O₂ into O₂ and H₂O (İpek et al. 2024). Given these enzymatic reactions, it becomes evident that the increase in the activities of SOD, POD, and CAT serves as an indicator that drought stress triggers an elevation in the activity of antioxidant enzymes. The increase in the contents of proline and soluble sugar enables crops to adapt to drought environments by osmotic regulation of redox balance, restoration of cell structure, and reduction of oxidative damage (Ghosh et al. 2022). MDA is often used to reflect the degree of damage to plant cell membranes (Zhou et al. 2023). Under drought stress, plants are known to accumulate excessive ROS (Fang and Xiong 2015), and the massive accumulation of ROS will cause destructive oxidation to nucleic acids, proteins, and lipids, which in turn leads to cell death (Mansoor et al. 2022). Due to its stronger antioxidant capacity, L1 can quickly remove excess H₂O₂ and reduce membrane lipid oxidation, thereby reducing the accumulation of MDA.

### 3.2 Changes in the Contents of Soluble Protein, Amylose and Total Starch in Proso Millet Under Drought Stress During the Germination Period

**FIGURE 2:**
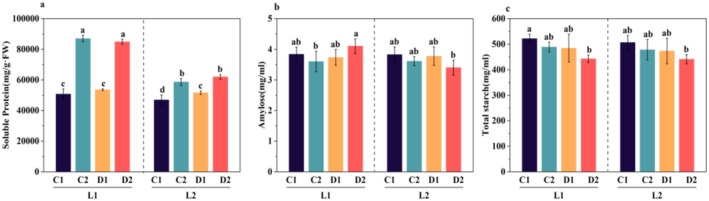
Changes in the contents of soluble protein, amylose and total starch in different proso millets at 24 h and 48 h under drought stress and normal germination. (a) soluble protein content; (b) amylose content; (c) total starch content; These values are the averages of three replicates. Vertical bars indicate the mean standard deviation. Significance is indicated according to Duncan's multiple range test (*p* < 0.05).

Drought stress during the germination period has a significant impact on the contents of soluble protein, amylose and total starch in proso millet. Compared with L1C1 and L1C2, the content of soluble protein in L1D1 and L1D2 increased significantly (Figure 2a). The change trend of L2 is consistent with that of L1. Compared with L1C1, the amylose content of L1D1 decreased slightly. Compared with L1C2, the amylose content of L1D2 increased significantly. With the increase of drought stress time, the amylose content of L1 showed a trend of first decreasing and then increasing (Figure 2b). Compared with L1C1, the total starch content of L1D1 decreased significantly. Compared with L1C2, the total starch content of L1D2 decreased significantly (Figure 2c). Compared with L2C1 and L2C2, the amylose content and total starch content of L2D1 and L2D2 decreased significantly. Consistent with the research of Hu et al. (Hu and Lu 2018), it may be that under drought stress, beta‐amylase (BAM) is up‐regulated. Proso millet copes with the energy shortage under water shortage conditions by strengthening starch catabolism and inhibiting anabolism (Cao et al. 2022). In order to maintain normal physiological activities, plants decompose and synthesize stored starch into soluble sugar (Subbarao, Chauhan, and Johansen 2000) to meet the plant's demand for carbon (Liu et al. 2024), and promote the growth of roots, buds and coleoptiles (Betts et al. 2017). These soluble sugars, By maintaining leaf turgor to enhance stomatal conductance for the efficient absorption of CO₂ and promoting the root's capacity to absorb more water (Wu et al. 2015), it can improve the cell's osmotic adjustment ability, reduce the damage to the structure and function of cell membranes, enzymes and proteins caused by osmotic water loss (Yang et al. 2017), and further enhance the drought resistance of proso millet during the germination period.

High amylose content leads to a fast starch gelation rate and high gel strength. In the processing and application of rice noodles, it can significantly reduce the breakage rate and loss rate of rice noodles, endowing the rice noodles with good cooking quality (Xiao et al. 2024). Meanwhile, amylose has low water‐holding capacity and poor water absorption but is expandable, with a relatively high gelatinization temperature and low calorie content. This makes it suitable not only for direct use in food processing but also as a food additive, playing an important role in the food industry (Mukti and Byars 2011). On the other hand, during the processing, soluble proteins can be decomposed into small molecular substances such as amino acids (Ban et al. 2018). The accumulation of these free amino acids and other substances helps to increase the nutritional content of proso millet. Soluble proteins have a significant impact on various functions of proteins. Generally, the higher the protein solubility, the higher its value. Therefore, the content of soluble proteins has also become a key index in food processing (Lyu et al. 2024).

### 3.3 Metabolic Analysis of Proso Millet Under Different Treatments

**FIGURE 3:**
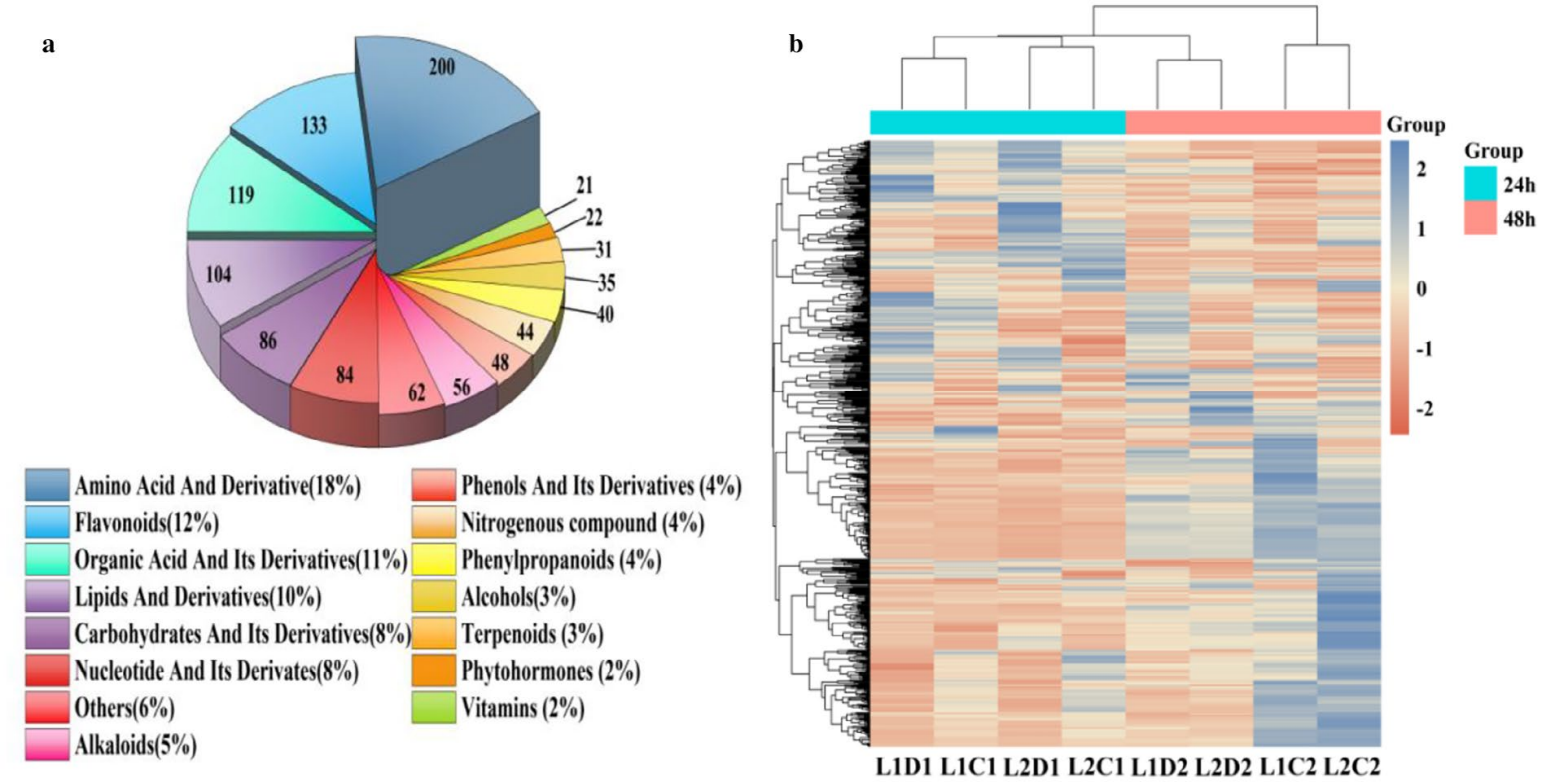
(a) Analysis of proso millet metabolite categories; (b) Hierarchical cluster analysis (HCA) of proso millet metabolism.

The total ion current (TIC) analysis of QC samples evaluated the reproducibility of the metabolite extraction and detection process (Figure S1). The same sample was identified multiple times, and its consistent retention time and peak intensity performance fully verified the stability and reliability of the analysis signal. In addition, we used the Pearson correlation coefficient to evaluate the data homogeneity within the repeat groups. The closer |r| is to 1, the higher the consistency between the data (Figure S2). Under the LC–MS/MS platform, using targeted‐like metabolomics technology, the changes in metabolites of two proso millet varieties with different drought tolerance were studied. A total of 1085 known metabolites were identified from 24 biological samples (Figure 3a, Table S1). These metabolites are divided into 15 categories, most of which are amino acids and derivatives (200 species, 18%), flavonoids (133 species, 12%), organic acids and derivatives (119 species, 11%), lipids and derivatives (104 species, 10%), sugars and derivatives (86 species, 8%), nucleotides and derivatives (84 species, 8%). The cluster heat map shows that proso millet under eight treatments can be divided into two groups: 24‐h treatment and 48‐h treatment (Figure 3b). Moreover, the metabolite contents in L1D2, L2D2 and L1C2, L2C2 are significantly different from the metabolite contents in L1C1, L1D1, L2D1 and L2C1, indicating that the metabolites of proso millet under different treatments are significantly different.

In this study, a total of 133 different types of flavonoids were found, including 70 flavonoids, 26 flavones and flavonols, 10 flavanones, 6 anthocyanins, 6 isoflavones, 1 catechin and its derivatives, 1 chalcone and dihydrochalcone, 1 xanthone and 12 other ketone compounds. Liu et al.'s research shows that germinated seeds affect germination by promoting the synthesis of flavonoids and inhibiting the synthesis of lignin (Liu et al. 2021). It may be that the synthesis of flavonoids helps to improve the antioxidant capacity of proso millet during germination (Santos et al. 2017), which is achieved by scavenging free radicals, chelating metal ions and regulating redox signaling pathways, helping to protect cells from oxidative stress damage, thereby delaying aging and preventing diseases (Cho et al. 2020; Liu et al. 2022). In addition, drought stress can promote an increase in the content of anthocyanins in proso millet (Ren, Liu et al. 2024). Anthocyanins can respond to biotic and abiotic stresses and scavenge oxygen free radicals to protect plants from damage by high‐density light (Fan et al. 2016).

Phenolics can have specific effects to influence plant hormone metabolism and enzyme systems and regulate plant growth (Wang, Sun, and Sun 2005). In this study, 48 phenolic compounds and their derivatives were found, including 9 phenolic acids (1‐O‐gentiinyl‐d‐glucose, 1‐caffeoylquinic acid, 2‐O‐caffeoylmaleic acid, 3,5‐dimethoxy‐4‐hydroxyphenol‐1‐O‐glucoside, 3‐O‐p‐coumaroylquinic acid, 6'‐O‐feruloyl‐d‐sucrose, p‐coumaric acid‐4‐O‐glucoside, rosmarinic acid‐3'‐O‐glucoside and sibiricose A3), expanding the diversity of phenolic compounds in cereals, which is consistent with the findings of Hao, Zhang, and Cao (2010). It may be that germination activates the activities of key enzymes and antioxidant enzymes in the synthesis of phenolic compounds (Li et al. 2024). At the same time, the release of precursor substances such as glucosides provides abundant substrates for the further synthesis of phenolic substances (Li et al. 2023).

Plants can reduce the impacts of drought stress through different amino acid metabolic pathways. Amino acids are the sources for the synthesis of various biofunctional macromolecular proteins. Under abiotic stress, plants can enhance their adaptability to adverse environments by regulating the activities of key enzymes and other means (Hildebrandt 2018). In this study, 200 amino acids and their derivatives were identified, accounting for 18% of all metabolites. The process of plant germination is accompanied by a series of physiological changes. Proteins are decomposed into amino acids under the action of enzymes, and some amino acids continue to participate in metabolism and interact with carbohydrates and lipids (Qin et al. 2024). Amino acids are one of the essential nutritional components for the human body. They not only provide nutrition but also some amino acids possess special flavors and make significant contributions to the taste of food. The composition and content of amino acids also determine the quality of food proteins (You et al. 2024).

### 3.4 Multivariate Analysis of Proso Millet Was Performed by PCA and PLS‐DA

**FIGURE 4:**
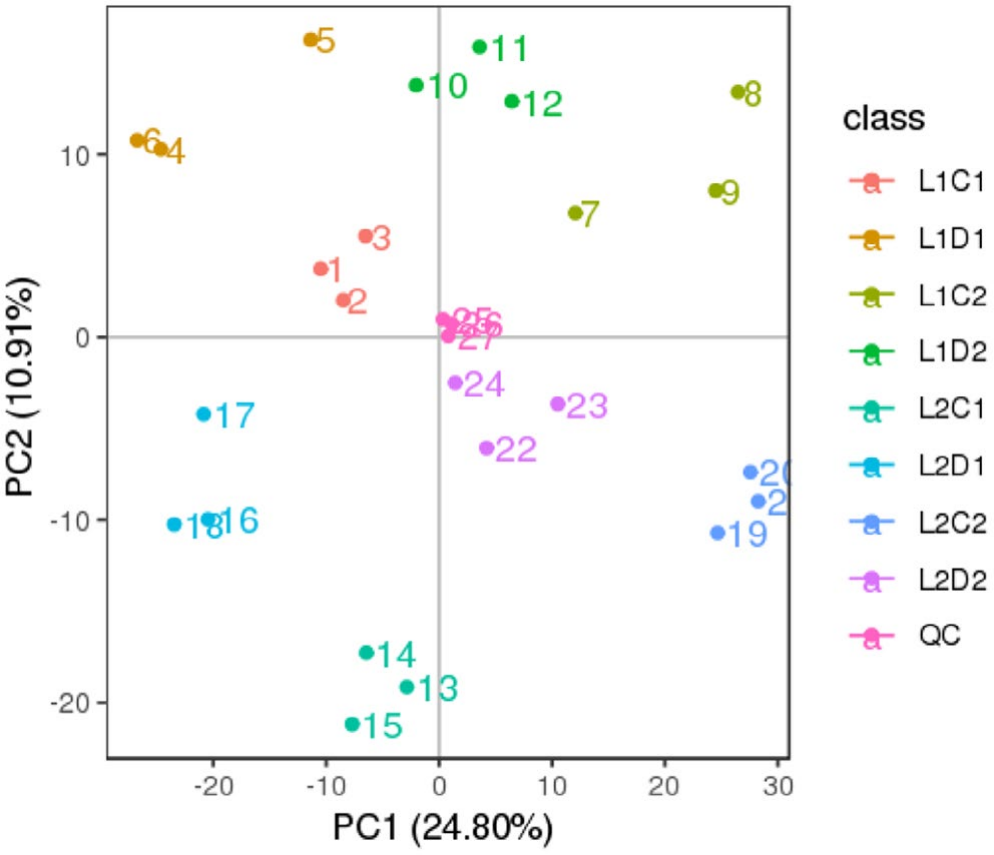
PCA analysis of metabolites in total samples of proso millet. The colored dots in the figure represent each sample, and the numbers are sample numbers.

Through PCA analysis of metabolites in proso millet during germination under different treatments (Figure 4), the results show that the combined contribution of the first two principal components (PC1 is 24.8% and PC2 is 10.91%) is 35.71%. In addition, QC samples are located in the center of the PCA plot. These results indicate that the metabolome has excellent stability. The PCA plot clearly shows the clear clustering of proso millet under various treatments, highlighting the metabolic differences between them. The tight clustering of replicates for each treatment emphasizes the repeatability of the experiment and further indicates the applicability of these results for subsequent qualitative and quantitative analyses.

**FIGURE 5:**
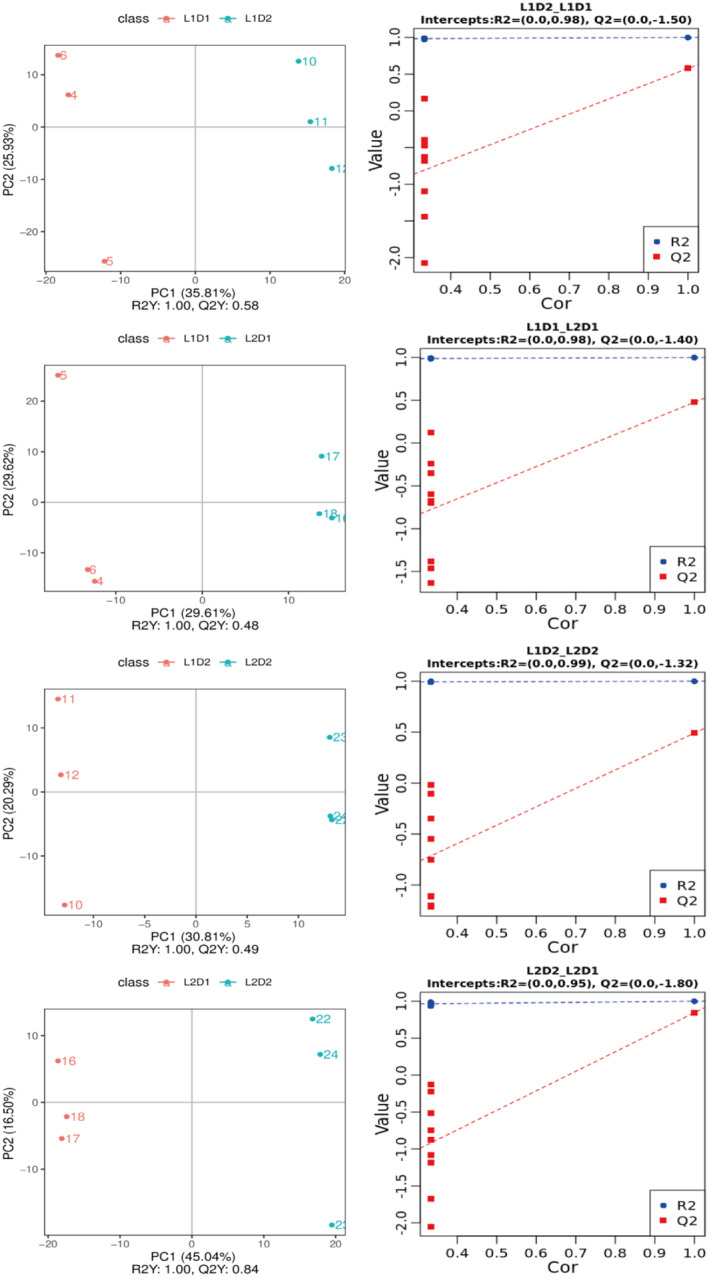
L1D1 vs. L1D2, L1D1 vs. L2D1, L1D2 vs. L2D2, and L2D1 vs. L2D2 PLS‐DA score plot and PLS‐DA ranking verification of proso millet.

Partial least squares discriminant analysis (PLS‐DA) was used to analyze the metabolites of proso millet during germination to determine the changes in metabolites at different treatments and germination times between two proso millet varieties, so as to construct a relationship model between metabolites and samples. *R* ^2^ *Y* is the interpretation rate and *Q* ^2^ *Y* is the prediction ability. When *R* ^2^ *Y* > *Q* ^2^ *Y*, it means that the model is well‐established. The PLS‐DA score plot shows (Figure 5, Figures S3 and S4) that *R* ^2^ *Y* > *Q* ^2^ *Y* is satisfied among all comparison groups, indicating that the model is well established. The verification of PLS‐DA model ranking shows that *R* ^2^ in each test sample group is greater than *Q* ^2^, and the intercept of the *Q* ^2^ regression line and the *Y*‐axis is < −0.05. This indicates that the samples detected in this study have good repeatability and stability and can be used for subsequent metabolic analyses such as screening differential metabolites.

### 3.5 Analysis of Differential Metabolic Profiles of Proso Millet

To identify the most important metabolites of two proso millet varieties during germination under different treatments, this study used the PLS‐DA model to calculate the variable importance in the projection (VIP) value between each group. Metabolites with VIP > 1, |log_2_FC| > 1, and P‐value < 0.05 are defined as differential metabolites (DEMs) (Table S2, Figure S5). Between L1C2 and L1C1, 128 DEMs were screened (110 up‐regulated and 18 down‐regulated). Between L2C2 and L2C1, 168 DEMs were screened (144 up‐regulated and 24 down‐regulated). Between L1C1 and L2C1, 44 DEMs were screened (36 up‐regulated and 11 down‐regulated). Between L1C2 and L2C2, 46 DEMs were screened (27 up‐regulated and 18 down‐regulated). Between L1D2 and L1D1, 47 DEMs were screened (31 up‐regulated and 16 down‐regulated). Between L2D2 and L2D1, 137 DEMs were screened (96 up‐regulated and 41 down‐regulated). Between L1D1 and L2D1, 42 DEMs were screened (12 up‐regulated and 30 down‐regulated). Between L1D2 and L2D2, 48 DEMs were screened (27 up‐regulated and 21 down‐regulated). Between L1D1 and L1C1, 64 DEMs were screened (17 up‐regulated and 47 down‐regulated). Between L1D2 and L1C2, 83 DEMs were screened (13 up‐regulated and 70 down‐regulated). Between L2D1 and L2C1, 85 DEMs were screened (30 up‐regulated and 55 down‐regulated). Between L2D2 and L2C2, 111 DEMs were screened (28 up‐regulated and 83 down‐regulated).

**FIGURE 6:**
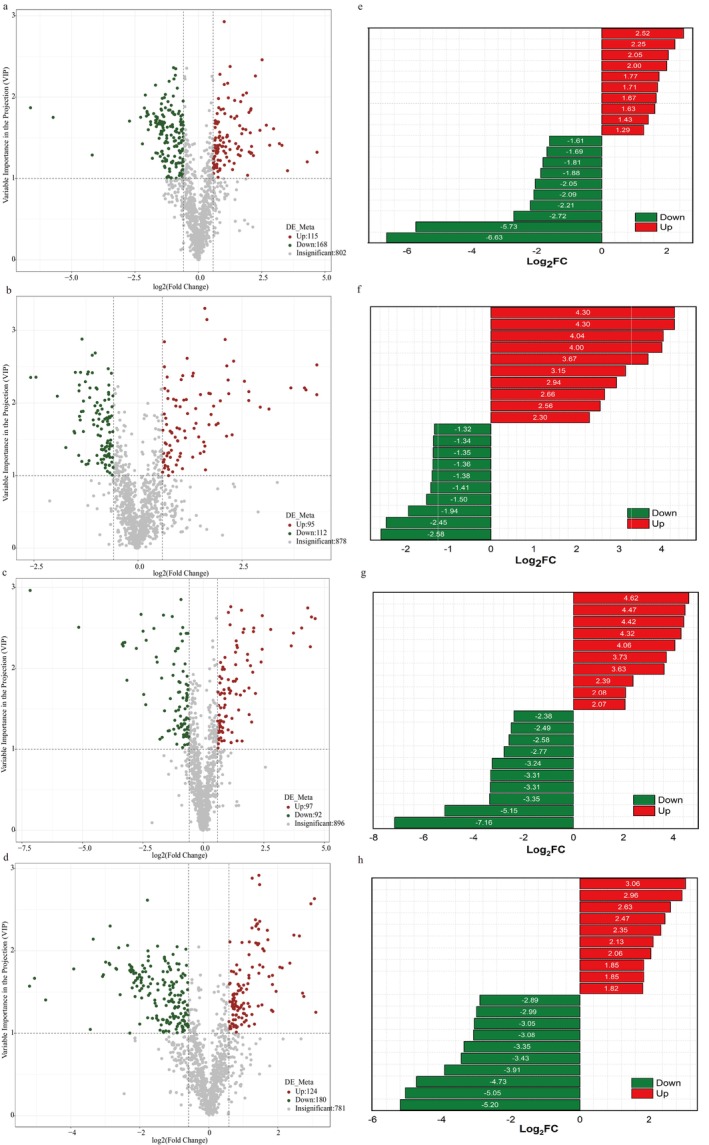
(a–d) Volcano plots show the differential expression levels of metabolites in proso millet samples. The abscissa is the VIP value, and the ordinate is the differential metabolites of each group. e‐h: The top 10 up‐regulated and down‐regulated differential metabolites in L1D1 vs. L1D2, L1D1 vs. L2D1, L1D2 vs. L2D2, and L2D1 vs. L2D2. The correct colors indicate the abundance of metabolites in different groups. Red has a relatively high content, and green has a relatively low content. The higher the VIP value, the greater the contribution to the discrimination of samples. Metabolites with VIP > 1 are defined as having significant differences.

According to the sorting from large to small of log_2_FC, the top 10 differentially expressed metabolites of up‐regulation and down‐regulation are marked respectively (Figure 6, Figure S6). In Figure 6e, the top 10 compounds with the largest up‐regulation between L1D1 and L1D2 are mainly sugars and flavonoids, and the top 10 compounds with the largest down‐regulation are mainly amino acids and their derivatives, plant hormones, lipids, vitamins, and nucleotides and their derivatives. In Figure 6f, the top 10 compounds with the largest up‐regulation between L1D1 and L2D1 are mainly amines, phenols, organic acids and their derivatives, and alcohols. The top 10 compounds with the largest down‐regulation are mainly phenylpropanoids, flavonoids, and terpenoids. In Figure 6g, the top 10 compounds with the largest up‐regulation between L1D2 and L2D2 are mainly organic acids and their derivatives, amines, phenolic acids and alcohols. The top 10 compounds with the largest down‐regulation are mainly alkaloids, flavonoids, phenylpropanoids and nitrogen‐containing compounds. In Figure 6h, the top 10 compounds with the largest up‐regulation between L2D1 and L2D2 are mainly amino acids and their derivatives, nitrogen‐containing compounds, amines and alkaloids. The top 10 compounds with the largest down‐regulation are mainly sugars, organic acids and their derivatives, flavonoids and amines. Between L1C1 and L1C2, the top 10 compounds with the largest up‐regulation are mainly amino acids and their derivatives, vitamins, plant hormones, nitrogen‐containing compounds and amines. The top 10 compounds with the largest down‐regulation are mainly sugars and their derivatives, flavonoids and lipids. Between L2C2 and L2C1, the top 10 compounds with the largest up‐regulation are mainly amino acids and their derivatives, amines and alcohols. The top 10 compounds with the largest down‐regulation are mainly sugars and their derivatives and flavonoids.

For the two varieties L1 and L2 of proso millet, during the normal germination process, there are relatively few differential metabolites at 24 h, and the metabolism is relatively stable. By 48 h, the number of differential metabolites increases and the up‐regulation trend is obvious. For variety L1, organic acids are almost up‐regulated, D‐(+)‐cellotriose in sugars is up‐regulated, and flavonoids and amino acids and their derivatives show complex changes. In variety L2, metabolites such as plant hormones, organic acids, and sugars change significantly. Some flavonoids are up‐regulated and some are down‐regulated. Amino acids and their derivatives also reflect the dynamic changes of protein metabolism. Different varieties show different metabolic response characteristics at 24 and 48 h, providing an important basis for understanding the physiological and metabolic mechanisms of proso millet germination.

### 3.6 Metabolic Pathway Analysis of Differential Metabolites During the Germination Period of Proso Millet

**FIGURE 7:**
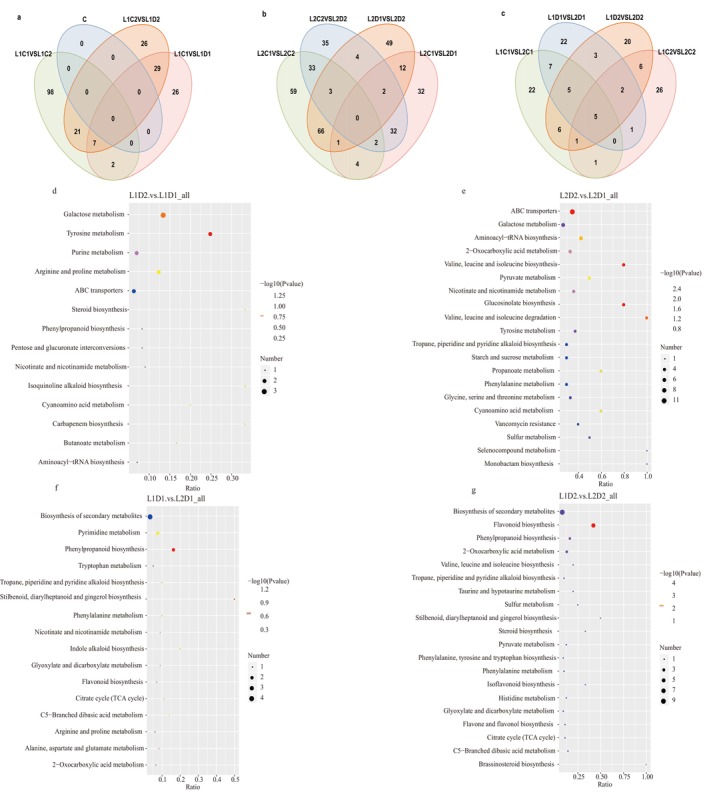
Venn diagram and pathway analysis of differential metabolite accumulation during the germination period of proso millet. (a) Variety L1; (b) Variety L2; (c) Variety L1 vs. Variety L2; (d‐g) KEGG pathway enrichment of differentially accumulated metabolites between each group (L1D2 vs. L1D1, L1D1 vs. L2D1, L2D2 vs. L2D1). Each bubble in the figure represents a metabolic pathway. The abscissa and bubble size together represent the magnitude of the influencing factors of this pathway. The larger the bubble, the greater the influence factor. The bubble color represents the *p*‐value of enrichment analysis. The darker the color, the higher the enrichment degree.

Venn diagram analysis shows that there are 80, 24, 18, and 22 unique metabolites between L1C1 vs. L1C2, L1C2 vs. L1D2, L1D1 vs. L1D2, and L1C1 vs. L1D1 of variety L1 respectively (Figure 7a), but there are no common differential metabolites in the four groups; between L2C1 vs. L2C2, L2C2 vs. L2D2, L2D1 vs. L2D2, and L2C1 vs. L2D1 of variety L2, there are 59, 35, 49, and 32 unique metabolites respectively (Figure 7b), and there are no common differential metabolites in the four groups, indicating that different treatment conditions have specific effects on the metabolism of proso millet. In the comparison between L1 and L2, there are 22, 22, 20, and 26 unique metabolites between L1C1 vs. L2C1, L1D1 vs. L2D1, L1D2 vs. L2D2, and L1C2 vs. L2C2 respectively (Figure 7c). There are 5 common differential metabolites in the four groups, namely 5,7‐Dihydroxy‐4‐methylcoumarin (Phenylpropanoids), 1‐Caffeoylquinic acid (Phenolic acid), Coniferylaldehyde (Alcohols), 1‐O‐Caffeoyl quinic acid (Phenolic acid), and L‐cysteine (Amino acids and derivatives).

KEGG enrichment analysis of DEMs was performed using the top 20 metabolic pathways with the smallest P‐value. Under normal germination (Figure S7a,d,g,h), the differential metabolites of proso millet are mainly enriched in pathways such as amino acid biosynthesis, biosynthesis of secondary metabolites, tyrosine metabolism, and ABC transporters. Under drought treatment (Figure S7d–g), the differential metabolites of proso millet are mainly enriched in the biosynthesis of secondary metabolites, tyrosine metabolism, and ABC transporter pathways. It may be because the increase in tyrosine metabolism and secondary metabolites can scavenge ROS and reduce lipid peroxidation of cell membranes (Tong et al. 2020; Sardari, Rezayian, and Niknam 2022) ABC transporters are involved in the transportation of ABA precursor substances and sugar metabolism (Yang et al. 2024). These pathways improve the drought tolerance of crops. The differential metabolites between L1D1 and L1C1 are mainly enriched in purine metabolism, amino sugar and nucleotide sugar metabolism, and starch and sucrose metabolism pathways (Figure S7b). The main enrichment pathways of differential metabolites between L1D2 and L1C2 are similar to those under drought for 24 h, but the pentose phosphate pathway is added (Figure S7c). The differential metabolites between L2D1 and L2C1 are mainly enriched in galactose metabolism, amino sugar and nucleotide sugar metabolism, and purine metabolism (Figure S7e). The differential metabolites between L2D2 and L2C2 are mainly enriched in the pentose phosphate pathway, tyrosine metabolism, and flavonoid biosynthesis pathway (Figure S7f). At 48 h of germination, the main enrichment pathways of differential metabolites all add the pentose phosphate pathway. It may be because when water is lacking, plants need more energy to maintain the basic physiological functions of cells. The pentose phosphate pathway can produce NADPH as a reducing force for biosynthesis during seed germination (Huang et al. 2003). The increase of NADPH will inhibit the production of ROS, thereby improving the antioxidant capacity of plants and protecting cells from drought damage (Kruger and Schaewen 2003).

### 3.7 Analysis of Significantly Enriched Pathways of Differential Metabolites During the Germination Period of Proso Millet

**FIGURE 8:**
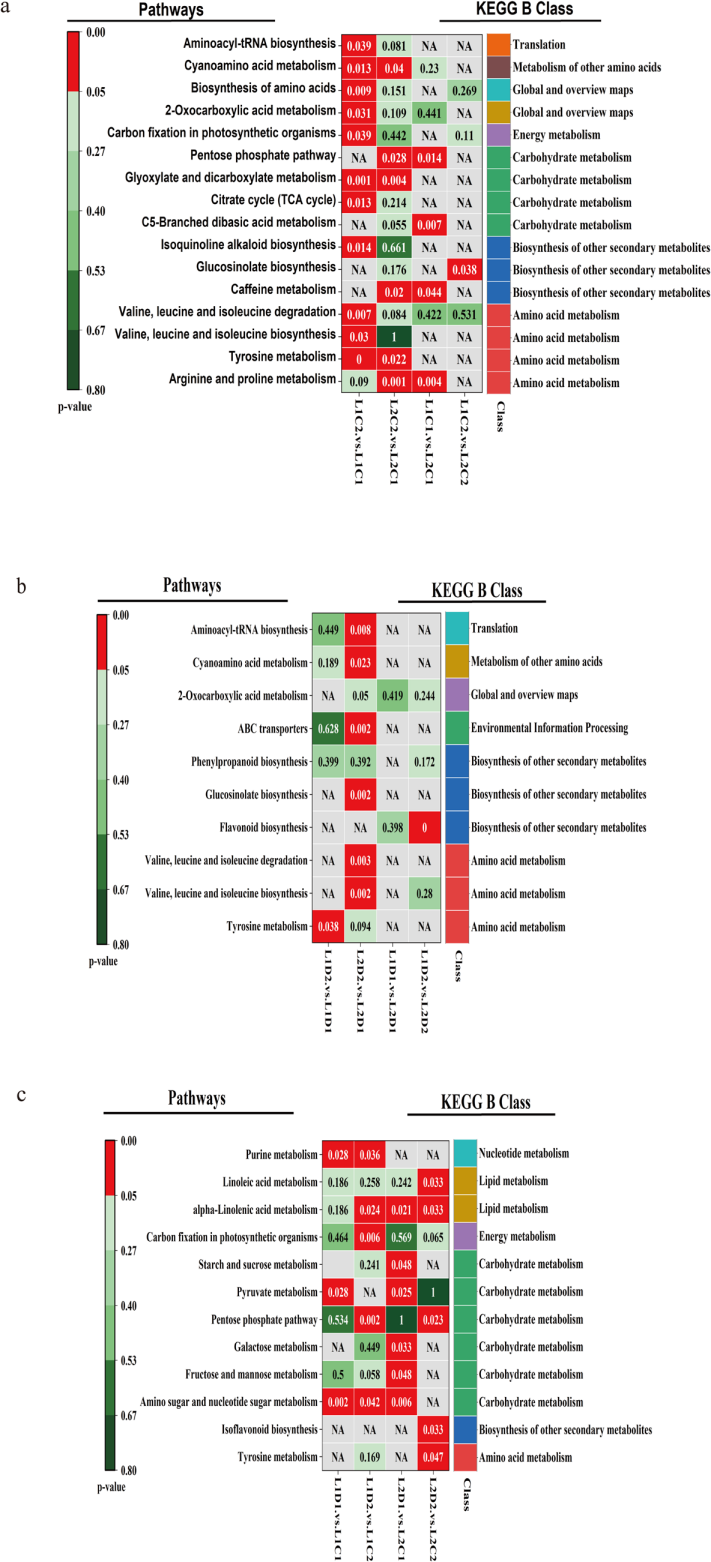
KEGG analysis of differential metabolites under different drought treatments during the germination period of proso millet. (a) KEGG analysis of metabolites based on *p*‐value under normal germination; (b) KEGG analysis of metabolites based on *p*‐value under drought treatment; (c) KEGG analysis of metabolites based on *p*‐value at different germination times. NA: Indicates that the differential metabolites in this group are not annotated to this pathway.

Further analysis was conducted on the significantly enriched (*p* < 0.05) pathways of differential metabolites. Under normal germination (Figure 8a, Table S3), between L1C2 and L1C1, DEMs annotated 38 KEGG pathways, and 11 pathways were significantly enriched, mainly involving amino acid metabolism, energy metabolism, sugar metabolism, and synthesis of other secondary products. In these pathways, 21 differential metabolites are involved, and the relative abundances of these substances are all up‐regulated. Between L2C2 and L2C1, DEMs annotated 45 KEGG pathways, and 6 pathways were significantly enriched, mainly involving sugar metabolism, amino acid metabolism, and synthesis of other secondary products. In these pathways, 12 differential metabolites are involved, and the relative abundances of these substances are all up‐regulated. Between L1C1 and L2C1, DEMs annotated 24 KEGG pathways, and 4 pathways were significantly enriched, mainly involving sugar metabolism, involving 5 differential metabolites (cis‐aconitic acid, citric acid, isocitric acid, (2S)‐2‐isopropylmalic acid, 2‐methylmalic acid), and the relative abundances of these substances are all up‐regulated. Between L1C2 and L2C2, DEMs annotated 17 KEGG pathways, and 1 pathway was significantly enriched as caffeine metabolism, involving the differential metabolite xanthosine, and the relative abundance of this substance was down‐regulated.

Under drought treatment (Figure 8b, Table S3), between L1D2 and L1D1, DEMs annotated 14 KEGG pathways, among which 1 pathway was significantly enriched, mainly the tyrosine metabolism in amino acid metabolism, involving 2 differential metabolites, maleic acid and tyrosine, and the relative abundances of these substances were all up‐regulated. Between L2D2 and L2D1, DEMs annotated 36 KEGG pathways, and 7 pathways were significantly enriched, mainly involving amino acid metabolism, sugar metabolism, and biosynthesis of other secondary metabolites. 17 differential metabolites were involved in these pathways, and among these substances, the relative abundances of 6 substances, namely (2S)‐2‐isopropylmalic acid, lactose, raffinose, maltotriose, maltose and melibiose, were down‐regulated, and the other 11 were all up‐regulated. Between L1D1 and L2D1, DEMs annotated 16 KEGG pathways, and there was no significantly enriched pathway (*p* < 0.05). The smallest *p* (0.06) was for phenylpropanoid metabolism. Among the 2 differential metabolites involved, the relative abundance of chlorogenic acid was up‐regulated and that of coniferyl aldehyde was down‐regulated. Between L1D2 and L2D2, DEMs annotated 22 KEGG pathways, and 1 pathway was significantly enriched, which was the flavonoid biosynthesis in the biosynthesis of other secondary metabolites. 5 differential metabolites were involved, namely hesperetin, chlorogenic acid, quercetin, naringenin, naringenin chalcone and butin. Among these substances, the relative abundance of chlorogenic acid was up‐regulated, and the other 4 were down‐regulated.

As can be seen from Figure 8c and Table S3, the DEMs between L1D1 and L1C1 annotated 29 KEGG pathways, among which 4 pathways were significantly enriched, mainly amino sugar and nucleotide sugar metabolism, galactose metabolism, starch and sucrose metabolism, and purine metabolism, mainly involving sugar metabolism and nucleotide metabolism. In these pathways, 11 differential metabolites are involved, and the relative abundances of uridine 5′‐diphosphate glucose, deoxyguanosine diphosphate, adenosine diphosphate, and xanthosine are up‐regulated, and the other 7 are all down‐regulated. The DEMs between L1D2 and L1C2 annotated 24 KEGG pathways, and 5 pathways were significantly enriched, mainly involving sugar metabolism, energy metabolism, and lipid metabolism. In these pathways, 13 differential metabolites are involved, and the relative abundances of ribitol, L‐(−)‐arabinitol, and xanthosine are up‐regulated, and the other 10 are all down‐regulated. The DEMs between L2D1 and L2C1 annotated 26 KEGG pathways, and 6 pathways were significantly enriched, mainly involving sugar metabolism and lipid metabolism. In these pathways, 13 differential metabolites are involved, and the relative abundances of uridine‐5′‐diphosphate galactose, uridine 5′‐diphosphate glucose, and (2S)‐2‐isopropylmalic acid are up‐regulated, and the other 10 are all down‐regulated. The DEMs between L2D2 and L2C2 annotated 27 KEGG pathways, and 5 pathways were significantly enriched, mainly involving sugar metabolism, lipid metabolism, biosynthesis of other secondary metabolites, and amino acid metabolism. In these pathways, 13 differential metabolites are involved, and the relative abundance of naringenin is up‐regulated, and the other 12 are all down‐regulated.

**FIGURE 9:**
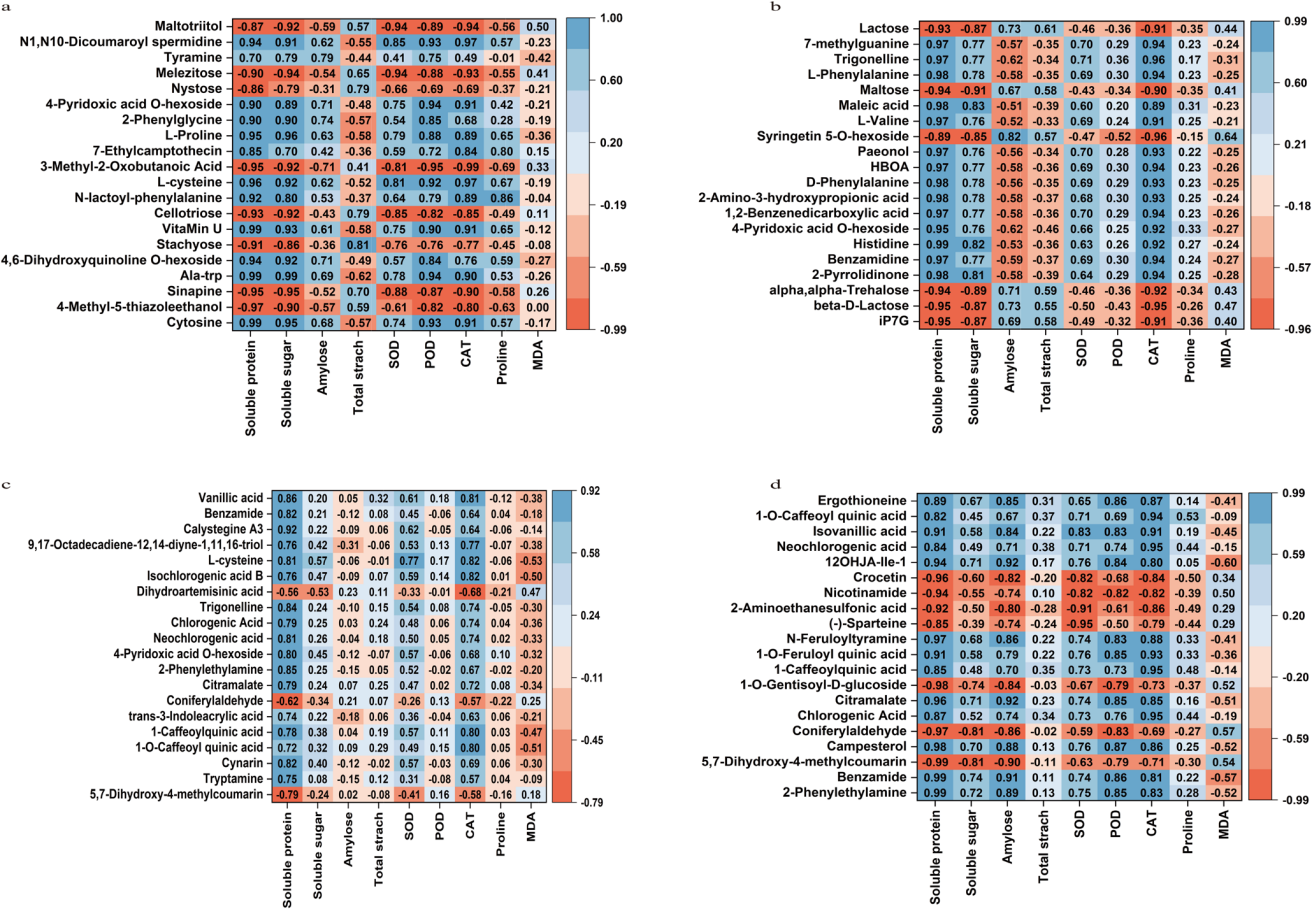
Correlation analysis between physiological indicators and top 20 DEMs during the germination period of proso millet. (a) L1D1 vs. L1D2; (b) L2D1 vs. L2D2; (c) L1D1 vs. L2D1; (d) L1D2 vs. L2D2.

Under different treatments, the metabolic pathways are mainly significantly enriched in sugar metabolism and lipid metabolism. Lipids are energy storage molecules, act as membrane components and play an important role in cell structure formation and signal transduction (Lim et al. 2015). The reason for lipid enrichment may be that when proso millet germinates, the expression level of LOX1 enzyme increases, catalyzing the infiltration of molecular oxygen into polyunsaturated fatty acids and producing oxidized lipids (He et al. 2022). These oxidized lipids not only act as signal molecules and participate in regulating the molecular response of cells to drought stress, but may also indirectly promote further enrichment of lipids by changing the lipid composition and physical properties of cell membranes. Lipids are the main components of cell membranes and store excess energy for the body to consume (Kang et al. 2024). The structural remodeling of phospholipids enhances cell membranes and provides better protection for germinating cells, enabling them to better tolerate adverse environments such as drought (Hou, Ufer, and Bartels 2016). It is found from Figure 9 that soluble protein, soluble sugar, amylose and antioxidant enzymes and proline in the L1D1VSL1D2 and L2D1VSL2D2 groups are negatively correlated with sugar metabolites such as Maltotriitol, Nystose, and Melezitose. This may be because soluble sugar and proline can osmotically regulate redox balance during drought (Ghosh et al. 2022), and the negative correlation with sugar metabolites may reflect the redistribution of plant sugar resources. During seed germination, sucrose provides energy for the growth of the germ (Lou, Wang, and Yu 2018). Sugar metabolism can accelerate the accumulation of hexose and provide a better material basis for plants to cope with adversity stress (Li, Tang et al. 2020), and can further enhance amylase activity and increase the content of total soluble sugar. At the same time, it significantly up‐regulates SAPK8/SAPK9 and enhances its drought tolerance during germination (Li et al. 2021).

As time goes by, the metabolic pathways and differential metabolites under normal germination have changed. Similarly, under drought treatment, there are also different metabolic characteristics between L1D1 and L1D2 and between L2D1 and L2D2. This indicates that the metabolic activities of proso millet at different germination time points are dynamically changing to meet the needs of growth. In the early stage of germination, it may be more focused on energy metabolism and amino acid metabolism. As the basic component of proteins, amino acids not only support various biosynthesis processes but also play an important role in cell signal transduction and stress adaptation (Shim et al. 2023). The reason for the enrichment of amino acid metabolism may be that plants sense drought signals, trigger osmotic imbalance and ROS accumulation, and simultaneously induce ABA synthesis (Xu and Xue 2024), while the amount of ABA can promote the synthesis and accumulation of amino acids (Asbahi, Maqtari, and Naji 2012). At the same time, branched‐chain amino acids are regarded as an important reserve for protein synthesis precursors in plants. Some proteins are degraded due to conformational changes, prompting plants to tend to accumulate more amino acids to reserve for future protein synthesis needs (Hildebrandt 2018), ensuring the smooth progress of various physiological activities during germination. As one of the basic amino acids, L—cysteine plays a crucial role in the biosynthesis of proteins and cell membranes and can effectively maintain the dynamic balance between the production and scavenging of reactive oxygen species in plants (Kang et al. 2020). Feng et al. (Feng, Yu, and Wang 2019) demonstrated that L—cysteine can reduce the accumulation of MDA and total phenolic content, thereby maintaining good color quality and nutritional value of food, achieving the effect of color protection and preservation and prolonging the shelf life. L—cysteine has strong reducibility and can reduce the disulfide bonds in gluten proteins to generate sulfhydryl groups, thus improving the extensibility of dough. When making noodles, adding an appropriate amount of L—cysteine can prevent the noodles from breaking easily (Pu et al. 2015).

In the later stage, it may be more focused on the synthesis of secondary metabolites. Plant secondary metabolites (SMs) are an important part of the plant defense system against pathogenic attacks and environmental stresses (Yang et al. 2018). According to chemical structure classification, SMs can be divided into phenolic acids, flavonoids, terpenoids, steroids and alkaloids, etc. (Santos et al. 2017). These secondary metabolites can protect plant cells from oxidative damage and improve drought resistance (Nguyen et al. 2016). In this study, the flavonoid content of two proso millet varieties decreased with the increase of germination time, which is consistent with previous studies (Naing and Kim 2021). The drought‐tolerant proso millet L1 consumes more flavonoids, which may be because flavonoids can better remove excess ROS and make its antioxidant defense ability stronger (Wang, Zhao et al. 2024). Flavonoids have various pharmacological and biological activities, including antioxidant, antitumor, antiviral and antibacterial properties (Kumar and Pandey 2013). Compared with the drought‐sensitive variety L2, the phenolic substances of the drought‐tolerant variety L1 first decreased and then increased. The main ones are chlorogenic acid, coniferyl aldehyde, N‐feruloyltyramine, etc. This is because the phenylpropanoid biosynthesis pathway will be activated under drought stress, leading to the accumulation of various phenolic compounds. These phenolic compounds can remove harmful reactive oxygen species and other effects and can cope with oxidative damage caused by drought (Sharma et al. 2019), ensuring the smooth germination of proso millet under drought conditions. The phenolic compounds synthesized during germination can promote antioxidant activity. Consuming fresh sprouts can provide antioxidant benefits similar to fresh blueberries (Bolívar and Luis 2010).

### 3.8 Correlation Analysis of Differential Metabolites and Physiological Indicators Under Drought Stress During Germination Period

We performed correlation analysis on the Pearson correlation coefficients between the top 20 DEMs (sorted from small to large *p* value) in each group and the Pearson correlation coefficients of physiological indicators. Under normal germination (Figure S8a,d,g,h), in L1C2 vs. L1C1, soluble sugar, soluble protein, SOD, CAT, and proline are negatively correlated with Sucralose, and the rest are positively correlated. Amylose content, total starch content, and MDA are the opposite. In L2C2 vs. L2C1, soluble sugar, soluble protein, SOD, CAT, and proline are negatively correlated with Raffinose, and the rest are positively correlated. Amylose and total starch are the opposite. In L1C1 vs. L2C1, soluble protein, total starch content, SOD, CAT, and MDA are negatively correlated with 5,7‐Dihydroxy‐4‐methylcoumarin, Coniferylaldehyde, D‐Gluconic acid, and D‐Galactonic acid, and the rest are positively correlated. In L1C2 vs. L2C2, soluble sugar, soluble protein, SOD, CAT are positively correlated with Chlorogenic Acid, 1‐Caffeoylquinic acid, and Narcissoside, and the rest are negatively correlated.

Under drought conditions (Figure 9), in L1D1 vs. L1D2, soluble protein, soluble sugar, amylose, SOD, POD, and CAT are negatively correlated with Maltotriitol, Nystose, Melezitose, 3‐Methyl‐2‐Oxobutanoic Acid, Cellotriose, Stachyose, 4‐Methyl‐5‐thiazoleethanol, and Sinapine, and the rest are positively correlated. In L2D1 vs. L2D2, soluble protein, soluble sugar, SOD, POD, CAT, and proline are negatively correlated with Lactose, Maltose, Syringetin 5‐O‐hexoside, iP7G, beta‐D‐Lactose, and alpha,alpha‐Trehalose, and the rest are all positively correlated. Amylose content, total starch content, and MDA are the opposite. In L1D1 vs. L2D1, soluble protein, soluble protein, SOD, and CAT are negatively correlated with Dihydroartemisinic acid, Coniferylaldehyde, and 5,7‐Dihydroxy‐4‐methylcoumarin, and the rest are all positively correlated. In L1D2 vs. L2D2, soluble protein, soluble sugar, amylose, SOD, POD, CAT, and proline are negatively correlated with (−)‐Sparteine, 2‐Aminoethanesulfonic acid, Nicotinamide, Crocetin, 1‐O‐Gentisoyl‐D‐glucoside, Coniferylaldehyde, and 5,7‐Dihydroxy‐4‐methylcoumarin, and the rest are all positively correlated.

Under different germination conditions and times (Figure S8b,c,e,f), in L1C1 vs. L1D1, soluble protein, soluble sugar, SOD, POD, CAT, and proline are negatively correlated with cis‐Aconitic acid and Adenosine 5'‐Diphosphate, and the rest are positively correlated. In L1C2 vs. L1D2, soluble protein, soluble sugar, SOD, POD, CAT, and proline are positively correlated with N‐Feruloyl‐3‐methoxytyramine, and the rest are negatively correlated. Amylose content, total starch content, and MDA are the opposite. In L2C1 vs. L2D1, soluble protein, soluble sugar, SOD, POD, CAT, and MDA are negatively correlated with N‐Feruloyl‐3‐methoxytyramine, Fmoc‐Ile‐OH, and Anthraquinone‐2‐carboxylic Acid, and the rest are positively correlated. In L2C2 vs. L2D2, soluble protein, soluble sugar, total starch, POD, CAT, and MDA are positively correlated with 1,7‐Dimethylxanthine and Allantoin, and the rest are negatively correlated.

## 4 Conclusion

In this study, through the comparison of drought‐tolerant proso millet L1 and drought‐sensitive proso millet L2under different drought treatments and at different times, it is clear that under drought treatment for 48 h, the activities of antioxidant enzymes such as SOD, POD, and CAT and the accumulation of osmotic adjustment substances such as proline, soluble sugar, and soluble protein in drought‐tolerant proso millet L1 are significantly higher than those under 24‐h treatment and significantly higher than those of drought‐sensitive proso millet L2. At 24‐h treatment, the metabolic pathways of L1 were mainly concentrated in carbohydrate and nucleotide metabolism, while those of L2 were mainly in carbohydrate and lipid metabolism. At 48 h, the metabolic pathways of L1 were mainly in carbohydrate, energy and lipid metabolism, and those of L2 were mainly in carbohydrate, lipid metabolism, biosynthesis of other secondary metabolites and amino acid metabolism. L1 contains a total of 144 differential metabolites, which are mainly involved in amino acids, lipids, sugar, and energy metabolism pathways. While there are a total of 175 differential metabolites in L2, mainly concentrated in sugar metabolism, lipid metabolism, biosynthesis of secondary metabolites and amino acid metabolism pathways. This study reveals that there are significant differences in the response mechanisms of different drought‐tolerant proso millets to drought stress during the germination period, providing key evidence for in‐depth understanding of the adaptation mechanism of proso millet to drought stress during the germination period and helping to further explore the physiological and metabolic regulation theory of plant germination under adversity.

## Author Contributions

**Mengyao Wang:** conceptualization (equal), data curation (lead), formal analysis (lead), methodology (equal), project administration (lead), resources (lead), supervision (lead), visualization (lead), writing – original draft (lead). **Yulu Hu:** conceptualization (equal), investigation (equal), methodology (equal), writing – review and editing (equal). **Jiao Mao:** investigation (equal), methodology (supporting), writing – review and editing (equal). **Yuanmeng Xu:** investigation (equal), methodology (equal), writing – review and editing (equal). **Shu Wang:** investigation (equal), methodology (equal), writing – review and editing (equal). **Lun Wang:** writing – review and editing (equal), funding acquisition. **Sichen Liu:** writing – review and editing (equal). **Zhijun Qiao:** conceptualization (equal), supervision (equal), writing – review and editing (equal). **Xiaoning Cao:** methodology (equal), visualization (equal), writing – original draft (supporting), funding acquisition.

## Ethics Statement

The authors have nothing to report.

## Conflicts of Interest

The authors declare no conflicts of interest.

## Supporting information


Figure S1.

Table S1.

Table S2.

Table S3.

## Data Availability

For access to the data utilized in this research, readers are encouraged to reach out to the corresponding author.
