# Supplementary material for: Physiological Responses and Metabolic Characteristics of Proso Millet Under Drought Stress During Germination Period
Source: Food Sci Nutr. 2025 Jan 31;13(2):e70001. doi: 10.1002/fsn3.70001 (PMC11782973; doi:10.1002/fsn3.70001)
Supplement: Supplementary file 1 — Figure S1. [file FSN3-13-e70001-s003.docx]

**Physiological responses and metabolic characteristics of proso millet under drought stress during germination period**

Mengyao Wang^a,b^, Yulu Hu^a,b^, Jiao Mao^a,b^, Yuanmeng Xu^a,b^, Shu Wang^a,b^, Lun Wang^a,b^, Zhijun Qiao^a,b^, Sichen Liu^a,b,*^, Xiaoning Cao^a,b,*^

^a^Center for Agricultural Genetic Resources Research, Shanxi Agricultural University, Taiyuan, 030031, China

^b^College of Agriculture, Shanxi Agricultural University, Jinzhong, 030801, China

^*^Corresponding Author:E-mail: caoxiaoning2008@163.com(X. Cao)

E-mail:[lsch209@163.com](mailto:lsch209@163.com)(S. Liu)


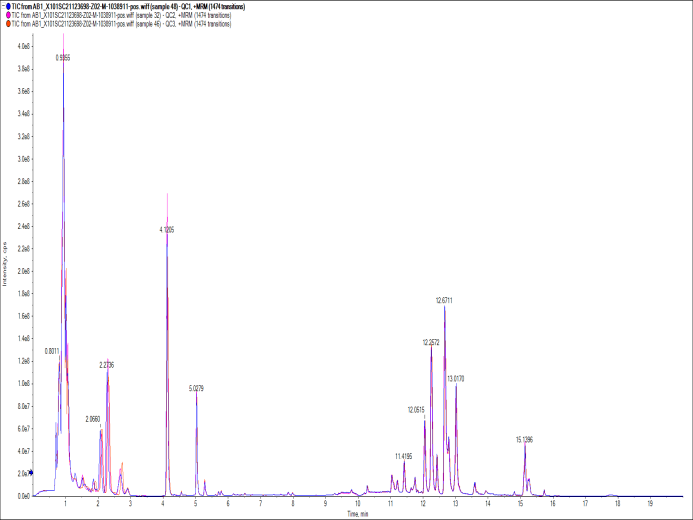

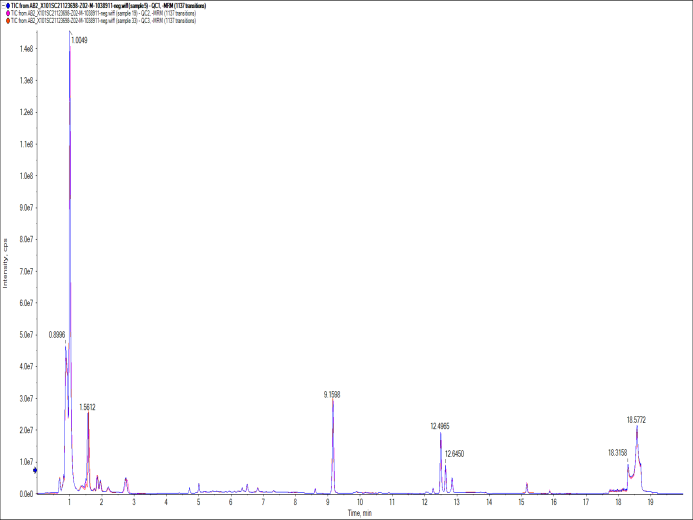


Fig.S**1** Total ion current (TIC) of QC samples, positive ion mode and negative ion mode


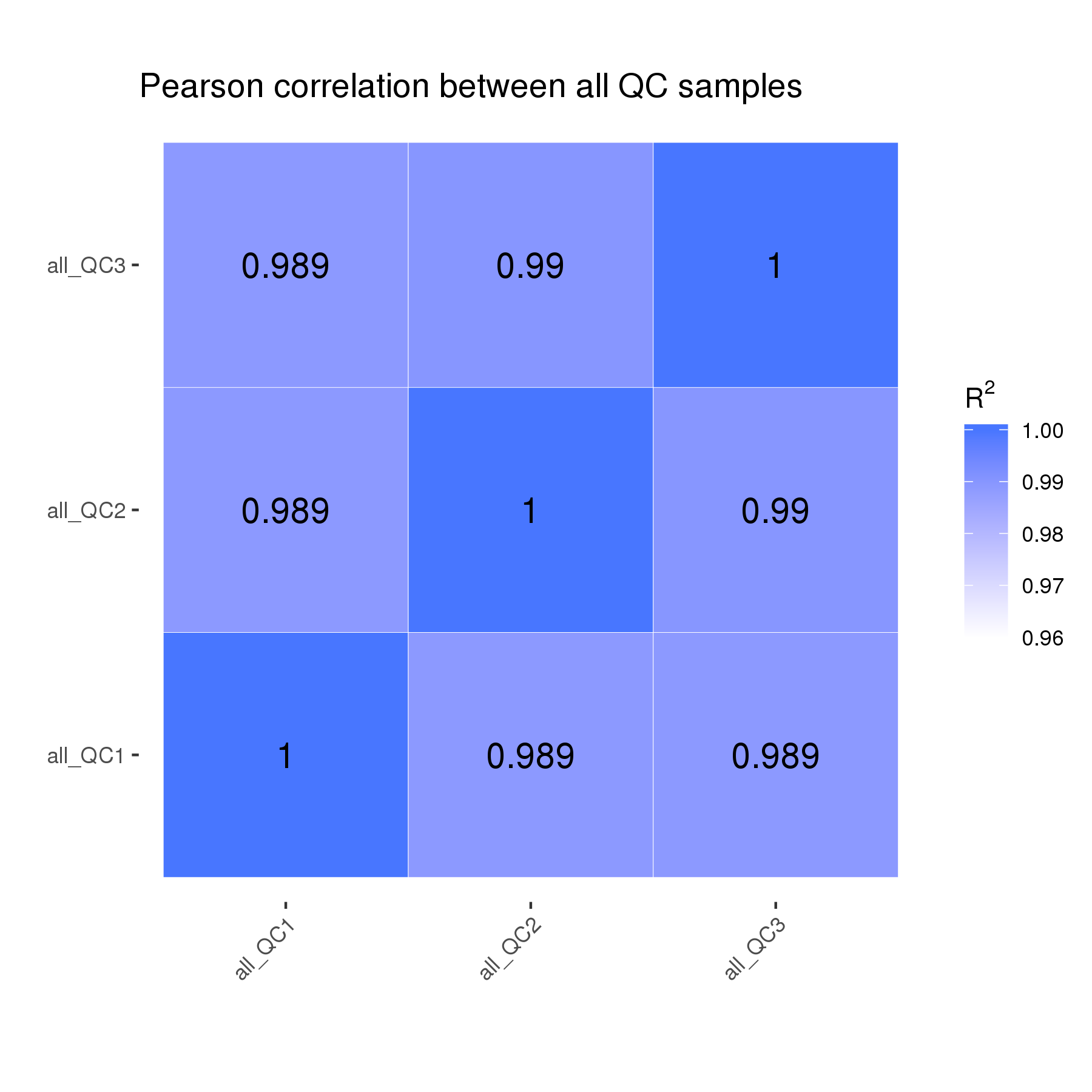


Fig.S**2** Person correlation coefficient

a b c


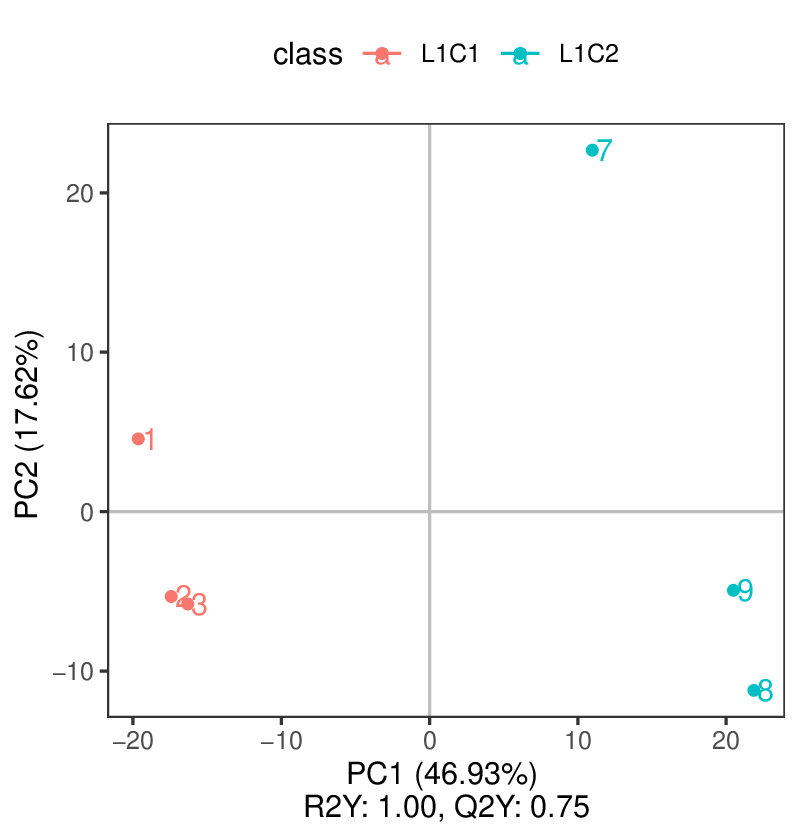

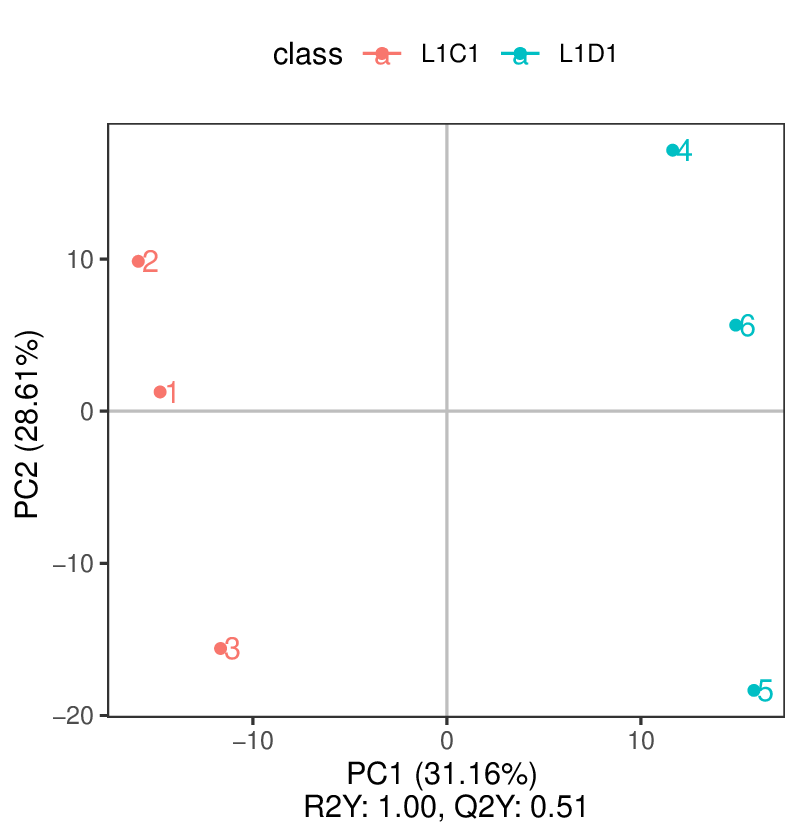

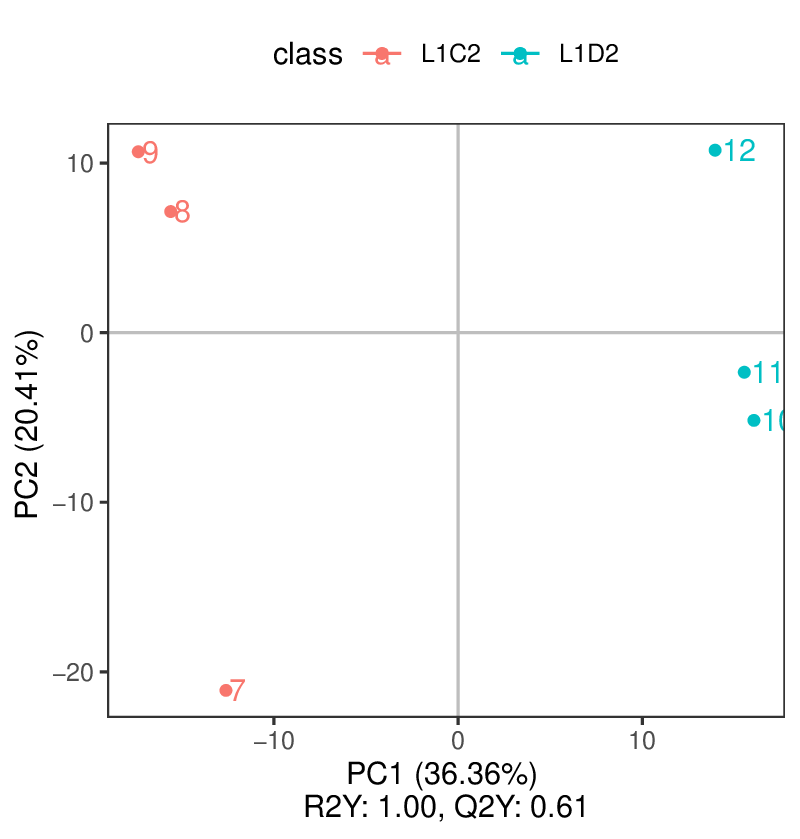


d e f


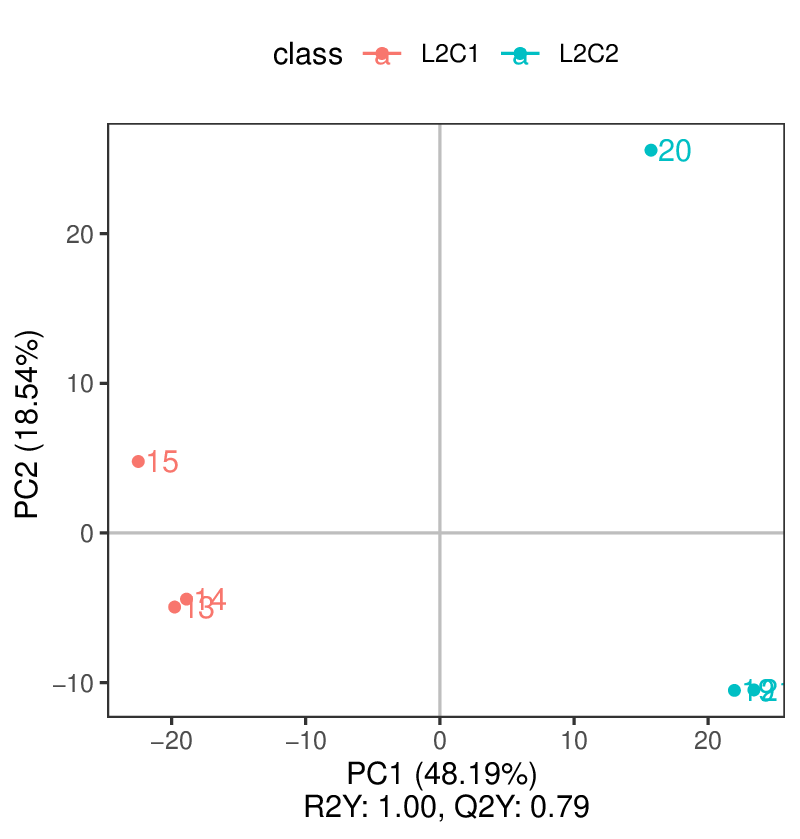

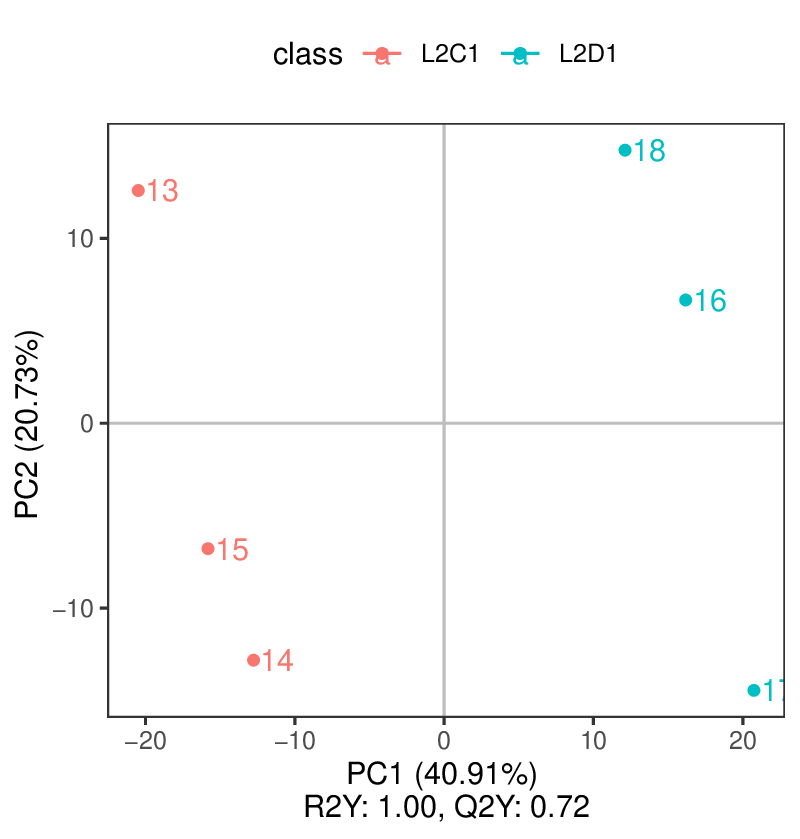

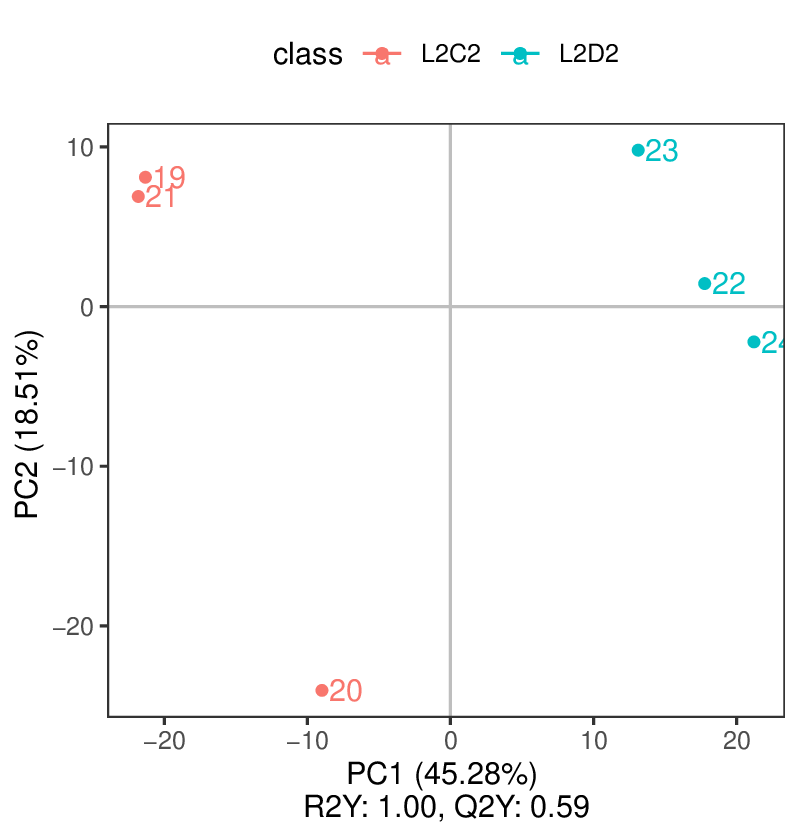


g h


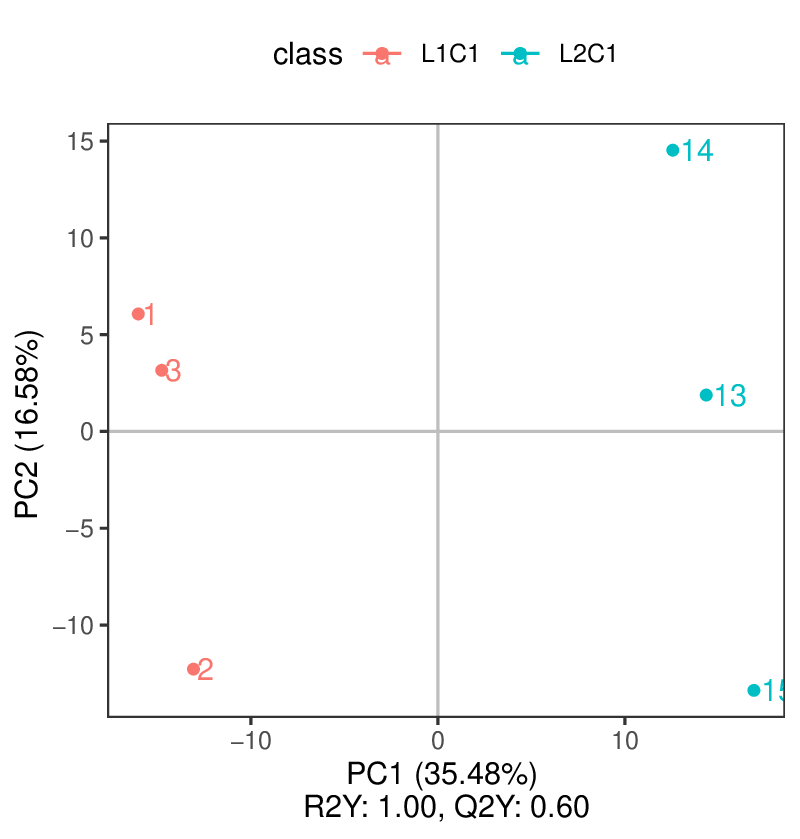

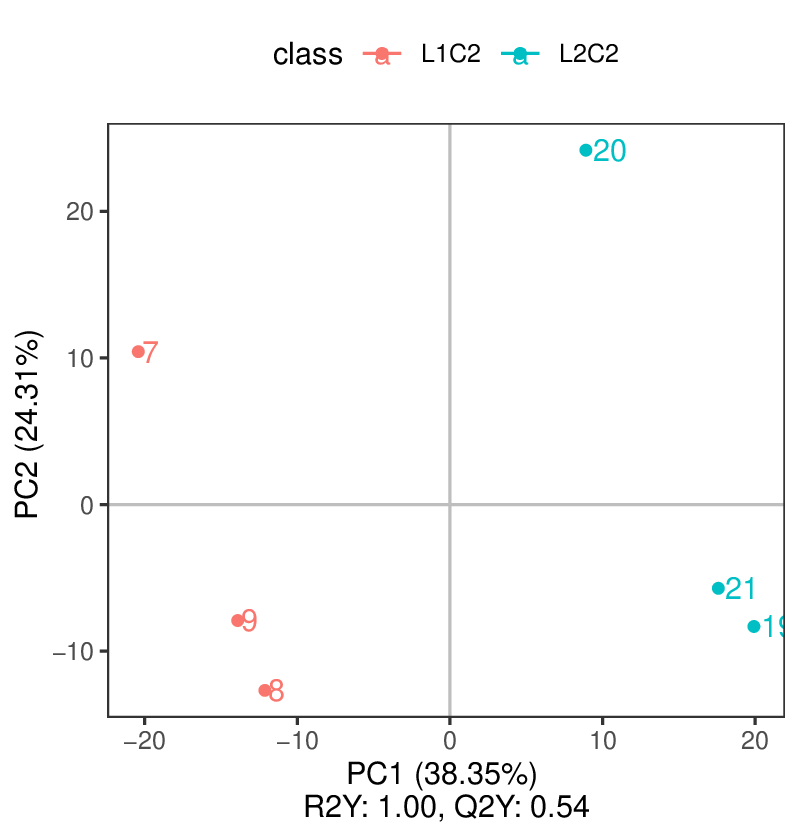


Fig.S**3** Partial least squares discriminant analysis (PLS-DA) scores of metabolites after different drought treatments in L1. (a) L1C2 vs L1C1. (b) L1C1 vs L1D1. (c) L1C2 vs L1D2. (d) L2C2 vs L2C1. (e) L2C1 vs L2D1. (f) L2C2 vs L2D2. (g) L1C1 vs L2C1. (h) L1C2 vs L2C2. The abscissa is the score of the sample on the first principal component; the ordinate is the score of the sample on the second principal component. R2Y represents the explanatory rate of the model, and Q2Y is used to evaluate the predictive ability of the PLS-DA model. When R2Y is greater than Q2Y, it indicates that the model is well established.

a b c


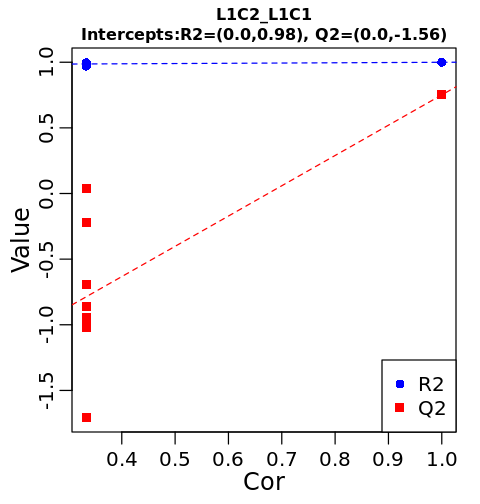

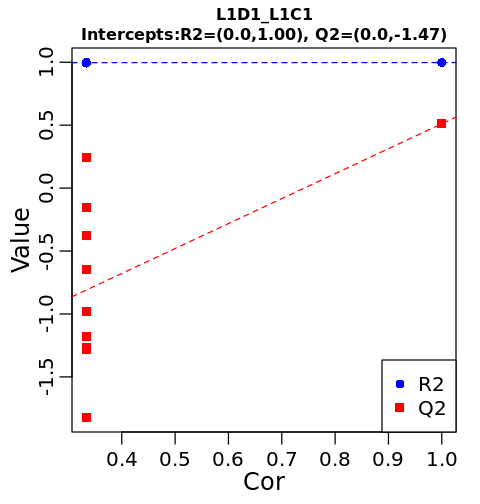

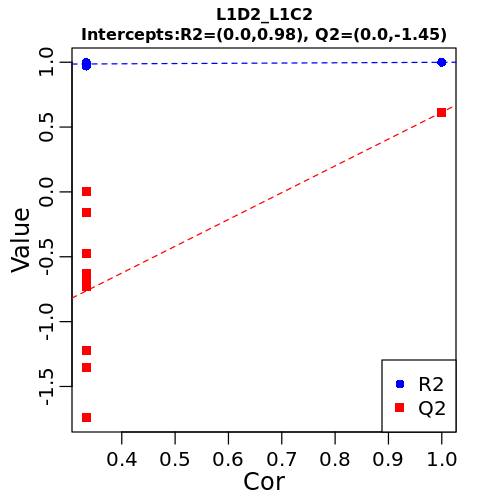


d e f


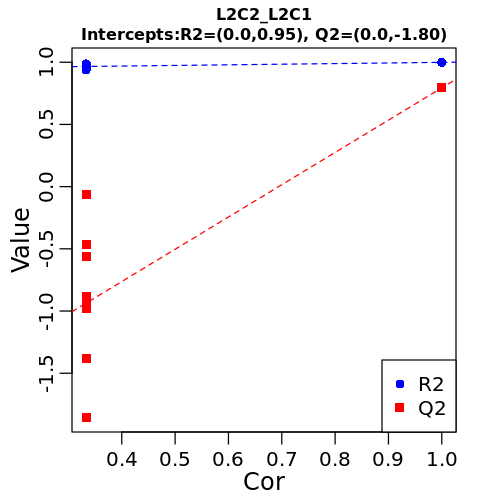

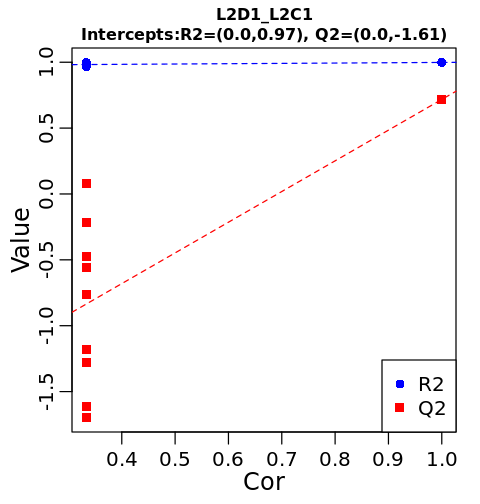

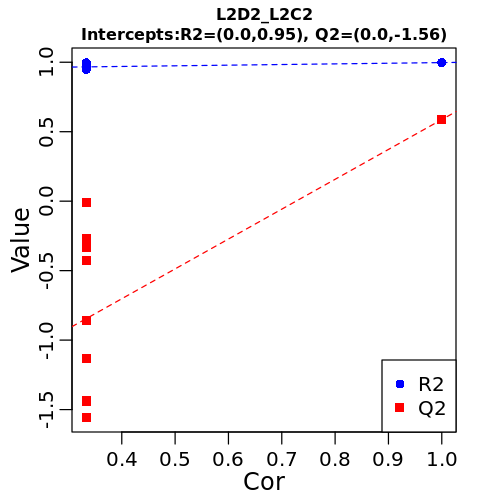


g h


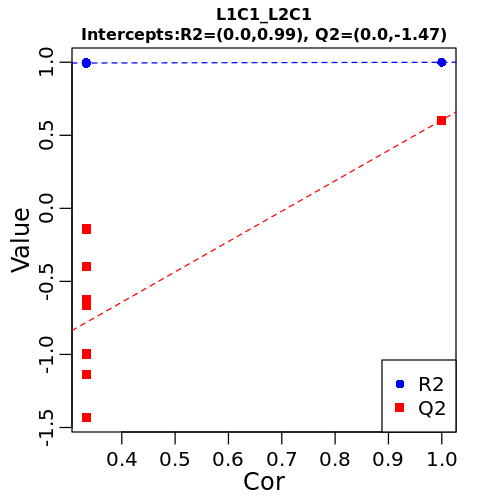

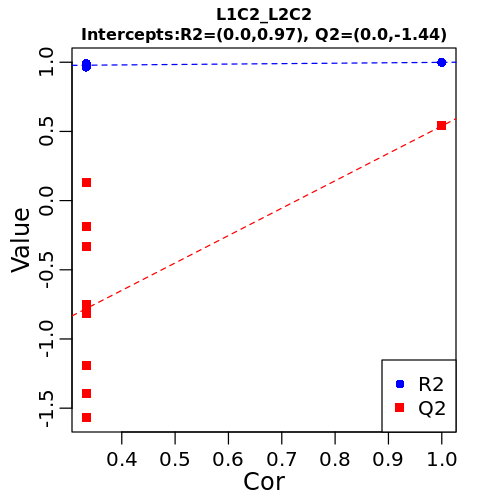


Fig.S**4** Permutation test random sorting method is used to evaluate the accuracy of the PLS-DA model. (a) L1C2 vs L1C1. (b) L1C1 vs L1D1. (c) L1C2 vs L1D2. (d) L2C2 vs L2C1. (e) L2C1 vs L2D1. (f) L2C2 vs L2D2. (g) L1C1 vs L2C1. (h) L1C2 vs L2C2. The abscissa represents the correlation between the randomly grouped Y and the original grouped Y, and the ordinate represents the scores of R2 and Q2; a point in the figure represents one test. The two points on the far right (x = 1.0) are the R2 and Q2 of the original model, and all the points on the left are the R2' and Q2' of the model after Y displacement. When the R2 value is greater than the Q2 value and the intercept of the Q2 regression line with the Y-axis is less than 0, it can be indicated that the model is not “overfitted”, which means that the model prediction results are reliable.

a b c


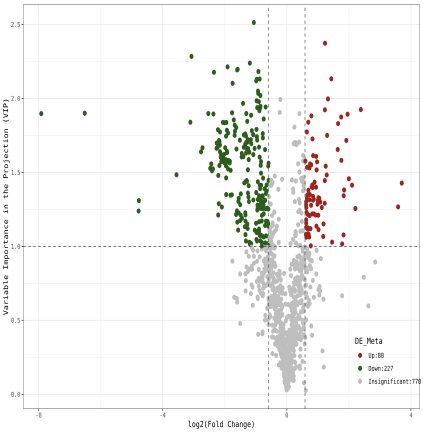

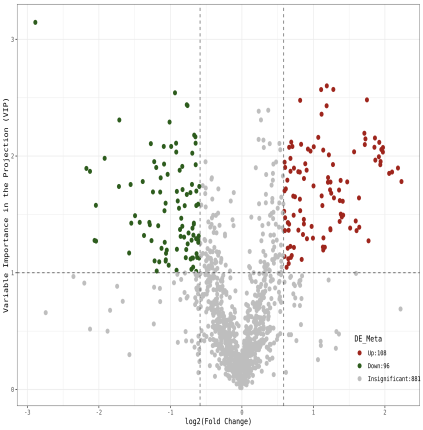

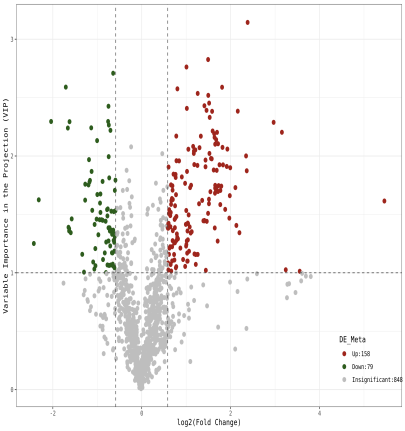


d e f


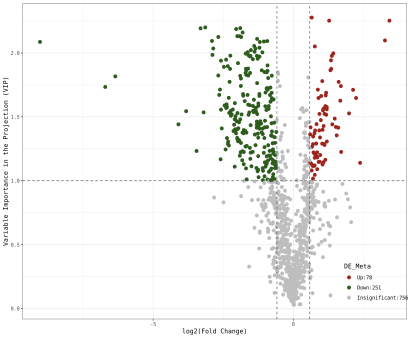

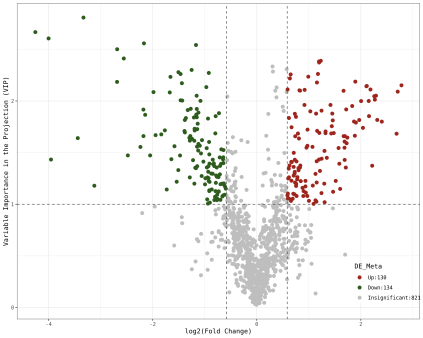

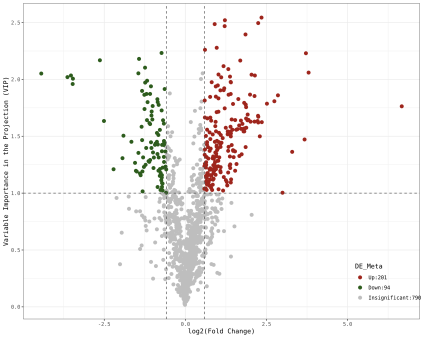


g h


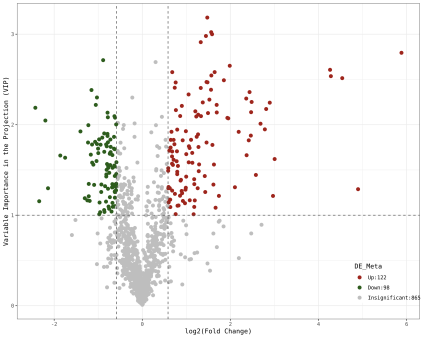

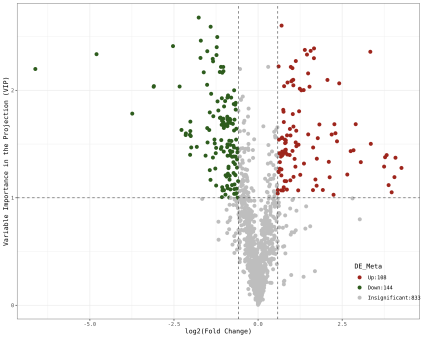


Fig.S**5** Volcano plots of differential metabolites after different drought treatments during the germination period of proso millet. (a) L1C2 vs L1C1. (b) L1C1 vs L1D1. (c) L1C2 vs L1D2. (d) L2C2 vs L2C1. (e) L2C1 vs L2D1. (f) L2C2 vs L2D2. (g) L1C1 vs L2C1. (h) L1C2 vs L2C2.

a b c


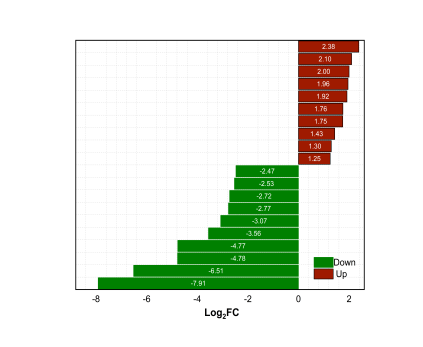

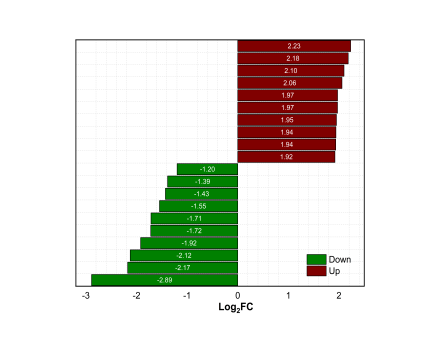

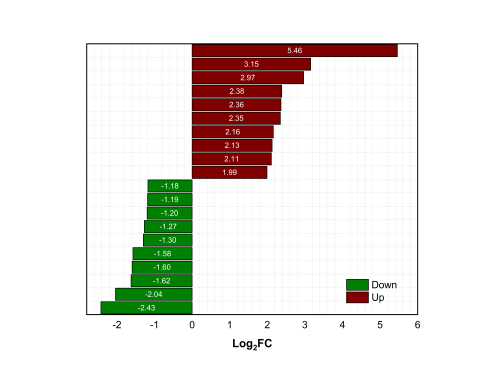


d e f


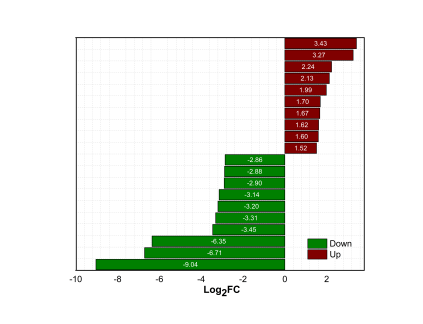

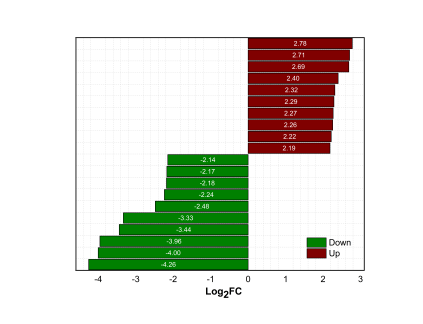

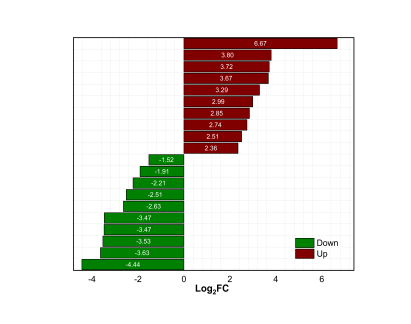


g h


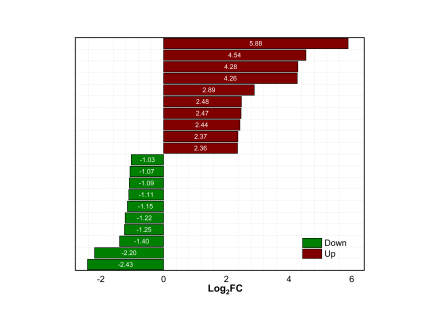

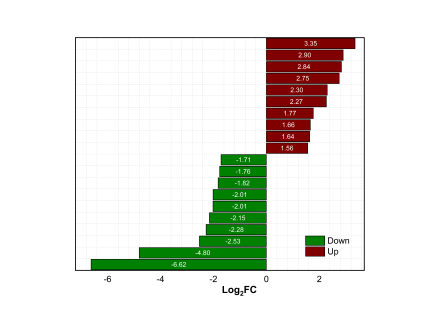


Fig.S**6** Top 10 up-regulated and top 10 down-regulated metabolites during the germination period of proso millet under drought stress. (a) L1C2 vs L1C1. (b) L1C1 vs L1D1. (c) L1C2 vs L1D2. (d) L2C2 vs L2C1. (e) L2C1 vs L2D1. (f) L2C2 vs L2D2. (g) L1C1 vs L2C1. (h) L1C2 vs L2C2.

a b c


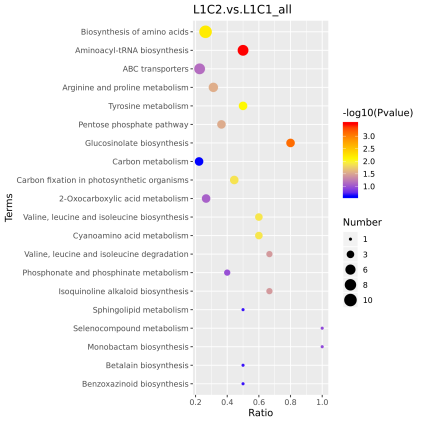

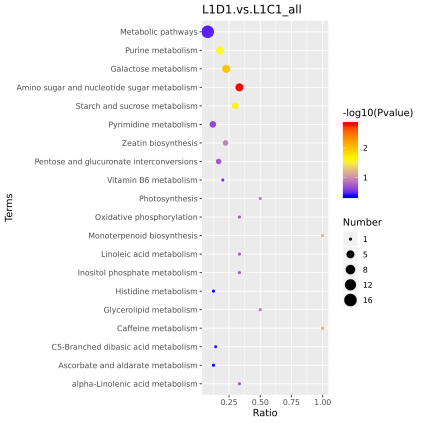

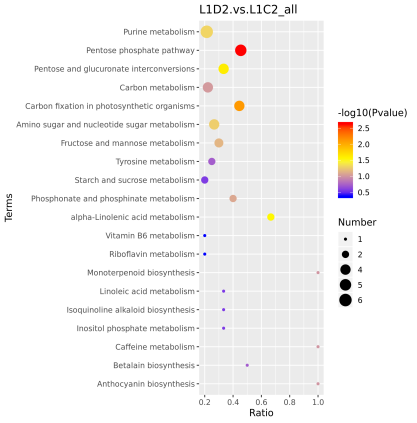


d e f


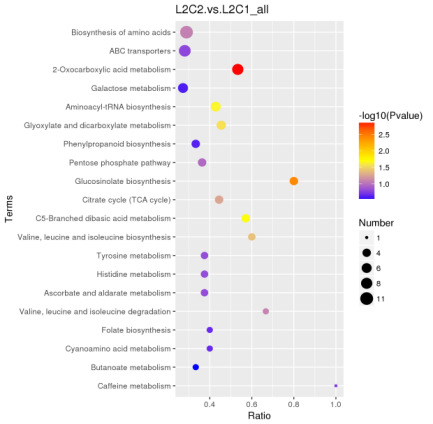

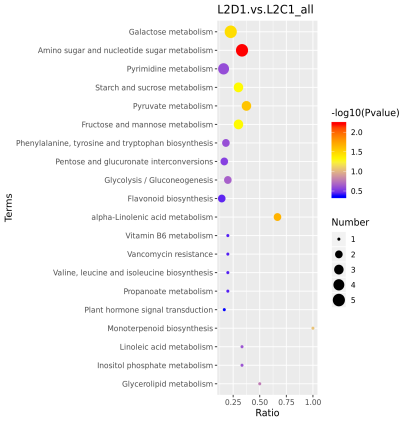

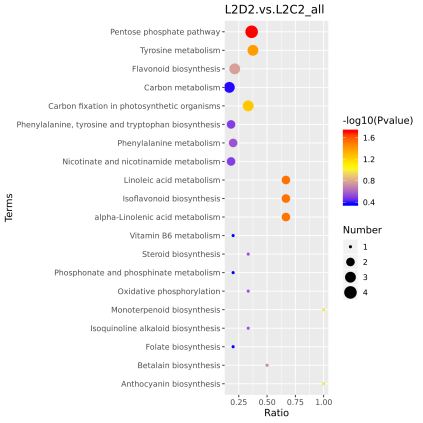


g h

**
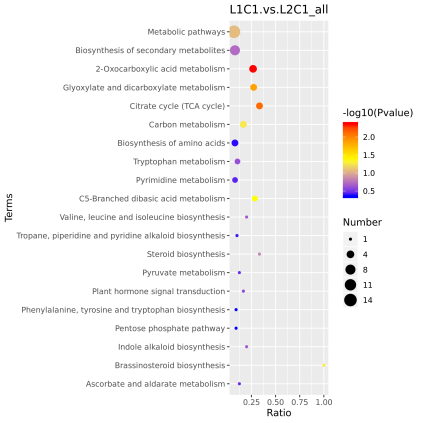

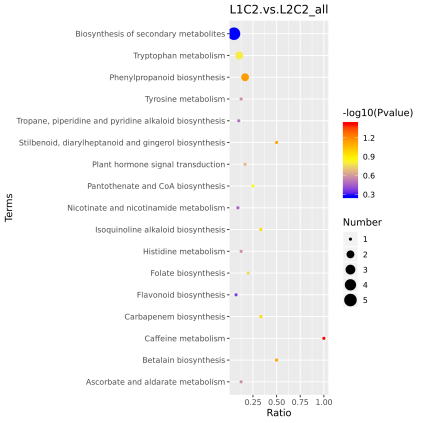
**

Fig.S**7** KEGG pathway enrichment of differentially accumulated metabolites between each group. (a) L1C2 vs L1C1. (b) L1C1 vs L1D1. (c) L1C2 vs L1D2. (d) L2C2 vs L2C1. (e) L2C1 vs L2D1. (f) L2C2 vs L2D2. (g) L1C1 vs L2C1. (h) L1C2 vs L2C2. Each bubble in the figure represents a metabolic pathway. The abscissa and the size of the bubble jointly represent the magnitude of the influencing factors of this pathway. The larger the bubble, the greater the influencing factor. The bubble color represents the p-value of enrichment analysis. The darker the color, the higher the enrichment degree.

a b c


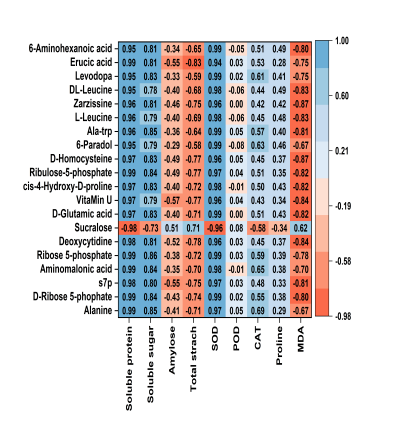

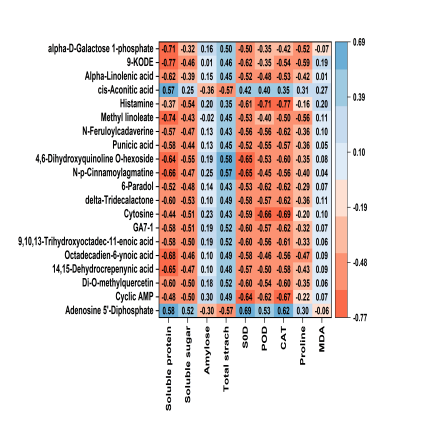

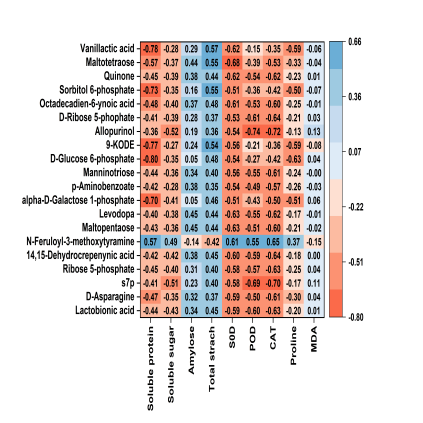


d e f


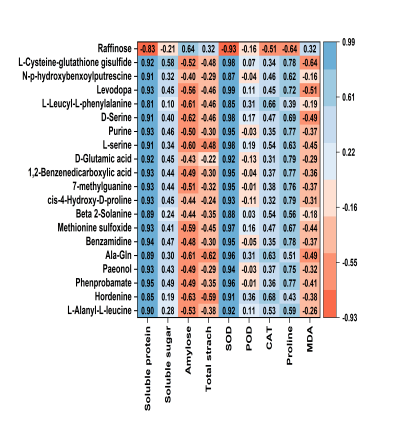

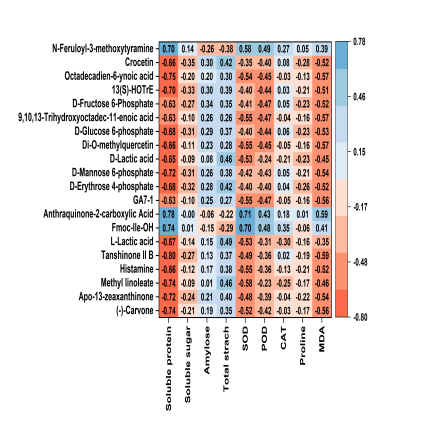

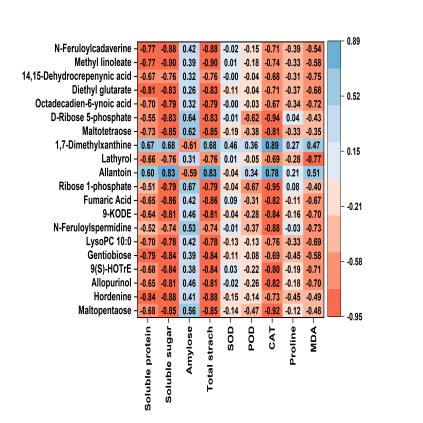


g h


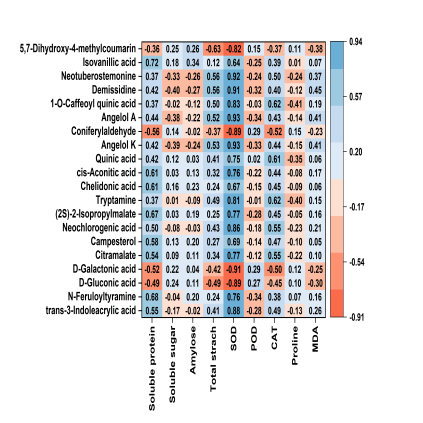

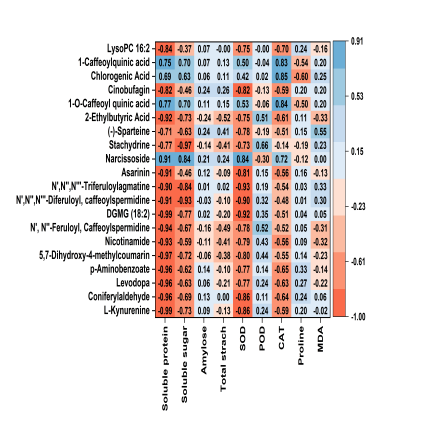


Fig.S**8** Correlation analysis between physiological indicators and top 20 DEMs during the germination period of proso millet.(a)L1C2vsL1C1.(b)L1C1vsL1D1.(c)L1C2vsL1D2.(d)L2C2vsL2C1.(e)L2C1vsL2D1.(f)L2C2vsL2D2. (g)L1C1vsL2C1. (h) L1C2vsL2C2.
